# Characterization of *Colletotrichum* Isolates from Strawberry and Other Hosts with Reference to Cross-Inoculation Potential

**DOI:** 10.3390/plants11182373

**Published:** 2022-09-11

**Authors:** Gunjan Sharma, Marcel Maymon, Vineet Meshram, Stanley Freeman

**Affiliations:** Department of Plant Pathology and Weed Research, Institute of Plant Protection, Agricultural Research Organization-Volcani Institute, Rishon LeZion 7505101, Israel

**Keywords:** anthracnose, morphology, multi-locus phylogeny, plant pathogenic fungi, taxonomy

## Abstract

*Colletotrichum* is an important phytopathogenic fungus that causes anthracnose disease in diverse agronomically important tropical food crops. Accurate pathogen identification is critical for early diagnosis and efficient management of anthracnose. ITS is not a reliable marker for this fungal genus due to its failure to phylogenetically resolve cryptic species. In this study, 36 *Colletotrichum* isolates belonging to the Acutatum, Boninense and Gloeosporioides species complexes were characterized using multigene phylogenetic analyses, morphology and pathogenicity assays. Additionally, the cross-inoculation potential of a representative subset of isolates was evaluated revealing that cross-infection potential is possible among the isolates belonging to the same species complex.

## 1. Introduction 

*Colletotrichum* (*Glomerellaceae*, *Glomerellales*, *Sordariomycetes*) is a ubiquitous and important plant pathogenic filamentous fungal genus [1]. *Colletotrichum* species have high genetic variability and diverse survival lifestyles according to their hosts and the environment [2,3,4]. Members of the species can exhibit endophytic, hemibiotrophic or necrotrophic lifestyles on their host plants [2,5]. Hemibiotrophy is a common mode of survival for this species, allowing an initial interaction with the host plant before infection takes place [6,7]. As a hemibiotroph, a quiescent infection is initiated via melanized appressoria that penetrate the host tissues to form infection hyphae, eventually leading to necrotrophy. Pathogenic *Colletotrichum* species usually exhibit hemibiotrophic and necrotrophic modes of survival and infection.

This genus is considered a “catalogue of confusion” [8], comprising more than 248 accepted species names [9]. Previously, the *Colletotrichum* species were named based on host-association, which created a lot of nomenclatural ambiguities. Due to the presence of morphologically similar cryptic species within each species complex, genealogical concordance for phylogenetic species recognition [10] is an important approach applied to designate and characterize a *Colletotrichum* species. *Colletotrichum* taxonomy has undergone multiple amendments based on the use of multi-locus molecular phylogenetic analyses [11,12,13,14,15,16,17,18]. Along with the genetic diversity of a fungal population, it is also fundamental to assess their pathogenic potential and epidemiology to understand completely the pathogenic nature of the fungi associated with a certain crop. Thus, the use of DNA-sequence data is vital in plant pathology, as it assists in monitoring plant health, pathogen risk assessment and devising pathogen management practices [19]. 

The *Colletotrichum* isolates used in this study were originally characterized based on the banding patterns obtained from RFLPs (restriction fragment length polymorphisms) and AP-PCRs (arbitrarily primed polymerase chain reactions) [20,21,22]. However, considering the recent nomenclatural updates in *Colletotrichum* taxonomy, in this study, we utilized current molecular phylogenetic analyses incorporating multi-gene sequence data, morphology and pathogenicity assay to reveal the species level of identification.

## 2. Results

### 2.1. Morphological Studies

Distinct morphological characteristics (colony color, growth rate, conidial measurements) were observed and recorded for representative isolates (*Colletotrichum godetiae*—ALM-KSH10, ALM-BZR82; *C. nymphaeae* –ANE-NL4, ANE-HV83C, STR-101, TUT-5954; *C. tamarilloi*—TOM-10; *C. acutatum*—ANE-NL12; *C. fioriniae*– APL2, ALM-US4; *C. karsti*—MAN76, *C. colombiense*—TOM6, PASS33; *C. brassicicola*– PASS65, *C. sydowii*—PASS35, *C*. *gloeosporioides s. l.*—APL7, Cg-Sc-A1) from 7-day-old cultures grown on PDA at 25 °C. Details regarding the morphological characterization of the isolates used in this study are presented in Table 1.

### 2.2. Phylogenetic Analyses and Assignment of Species

The 36 representative *Colletotrichum* isolates used in this study were distributed to their respective species complex on the basis of the ITS sequence data and corresponding NCBI-BLAST result. Twenty-four *Colletotrichum* isolates were assigned to the *C. acutatum* species complex, eight *Colletotrichum* isolates were assigned to the *C. boninense* species complex, and three *Colletotrichum* isolates were assigned to the *C. gloeosporioides* species complex. One isolate originally isolated from passiflora in Colombia, was found to be related to *C. sydowii*, which is a singleton species with limited reference sequence data. Thus, the identity of PASS35 according to the percent sequence identity of the ITS, *act*, *chs1*, *gapdh*, *his3* and *tub2* sequences corresponds to the type strain sequences of *C. sydowii*. The phylogenies of the three isolates belonging to the *C. gloeosporioides* species complex have been described in the supplementary information (Supplementary Figure S1).

### 2.3. Phylogenetic Analyses of the Isolates Belonging to the C. acutatum Species Complex

**Figure 1:**
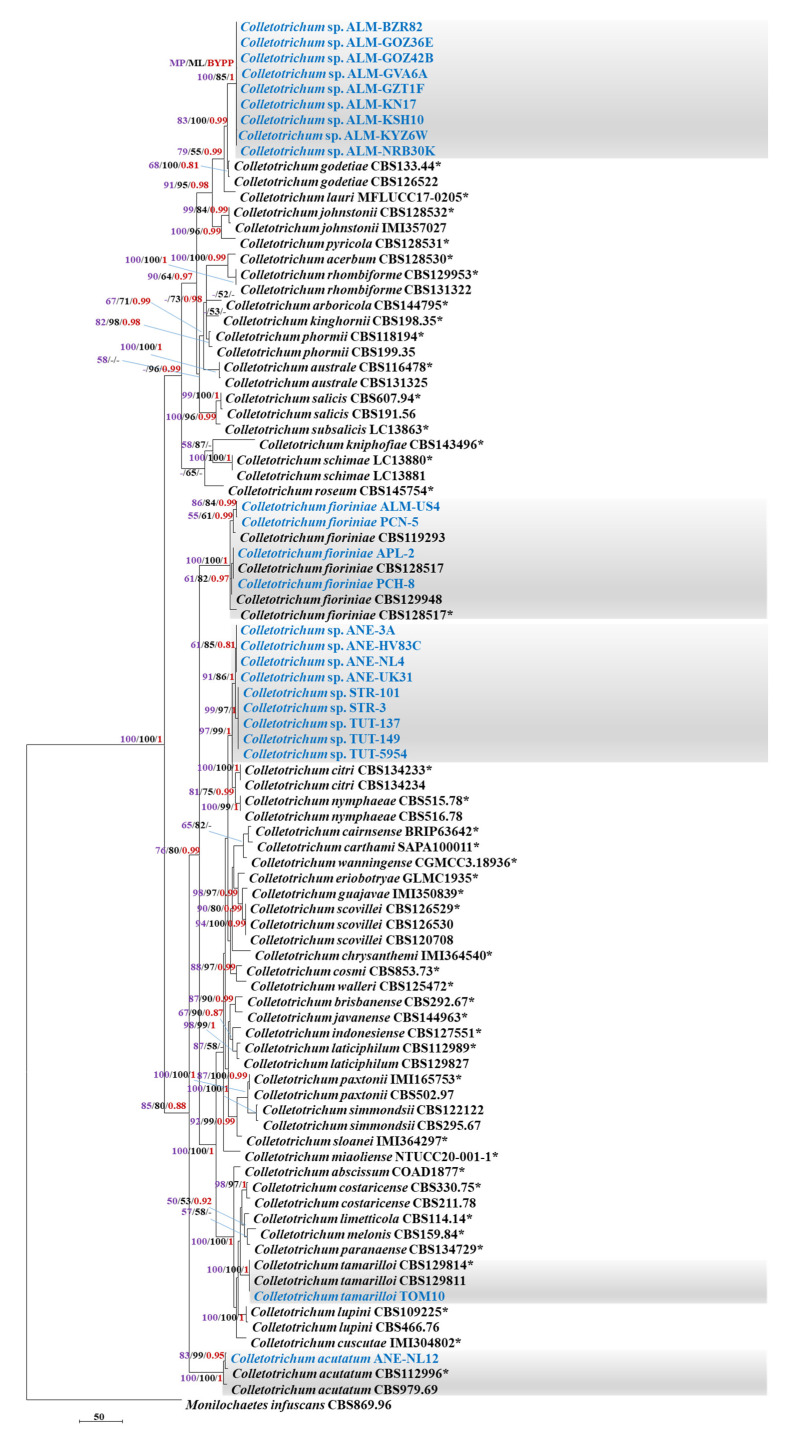
First of the 147 equally most parsimonious trees obtained from a heuristic search of the combined ITS, *act*, *chs1*, *gapdh*, *his3* and *tub2* sequence alignment of the *Colletotrichum* isolates in the Acutatum complex. The MP and ML bootstrap support values (>50%) and Bayesian posterior probabilities (>0.90) are displayed at the nodes (ML/MP/PP). The tree was rooted to *Monilochaetes infuscans* (CBS 869.96). The bar indicates 50 changes. The isolates from this study are highlighted in blue. (* = Type strain).

A concatenated dataset of six genes (ITS, *act*, *chs1*, *gapdh*, *his3* and *tub2*) was used for the phylogenetic analyses of 24 *C. acutatum* species complex members used in this study. *Monilochaetes infuscans* (CBS 869.96) was used as the outgroup, and 65 species in the Acutatum complex were used as a reference in the analysis. The multigene sequence alignment contained 2173 characters [ITS: 1–554; *act*: 555–817; *chs1*: 818–1094; *gapdh*: 1095–1368; *his3*: 1369–1755; *tub2*: 1756–2173], including gaps. Sixty ambiguous characters were excluded from the alignment, and, of the remaining 2113 included characters, 1431 characters were constant; 283 variable characters were parsimony-uninformative; and 399 characters were parsimony-informative. The parsimony analysis yielded 147 trees that were equally the most parsimonious; the topology of one is shown in Figure 1 [tree length (TL) = 1285, consistency index (CI) = 0.661, retention index (RI) = 0.905, rescaled consistency index (RC) = 0.598, homoplasy index (HI) = 0.339]. The Bayesian analysis of the combined alignment lasted 5,000,000 generations, and the resulting trees were used to calculate the posterior probabilities. The bootstrap support values of the MP analysis (MP > 50%), ML analysis (ML > 50%) and the BI posterior probabilities (PP > 0.90) are depicted at the branch nodes (Figure 1). 

Based on the MP and ML phylogenetic analyses (Figure 1), the ANE-NL12 (anemone) isolate clustered with the ex-type sequences of *C. acutatum sensu stricto* CBS112996 (MP 100%, ML 100%, PP 1.0); PCN5 (pecan), PCH8 (peach), APL2 (apple), ALM-US4 (almond) clustered with the ex-type sequences of *C. fioriniae* CBS128517 (MP 100%, ML 100%, PP 1.0); and TOM10 (tamarilla) clustered with the ex-type sequences of *C. tamarilloi* CBS129814 (MP 100%, ML 100%, PP 1.0). 

**Figure 2:**
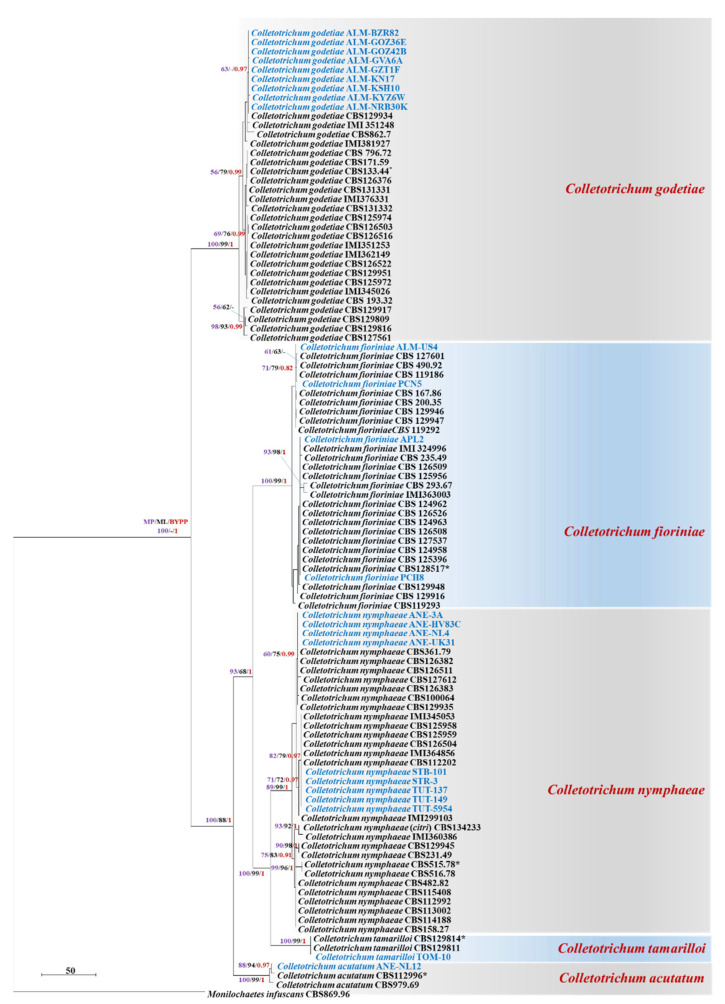
First of 205 equally most parsimonious trees obtained from a heuristic search of the combined ITS, *act*, *chs1*, *gapdh*, *his3* and *tub2* sequence alignment of the representative *Colletotrichum* isolates belonging to the *C*. *godetiae*, *C*. *nymphaeae*, *C. tamarilloi*, *C. fioriniae* and *C*. *acutatum* clades. The MP and ML bootstrap support values (>50%) and Bayesian posterior probabilities (>0.90) are displayed at the nodes (ML/MP/PP). The tree was rooted to *Monilochaetes infuscans* (CBS 869.96). The bar indicates 50 changes. The isolates from this study are highlighted in blue. (* = Type strain).

However, the isolates from almond, anemone and strawberry were placed within or next to the clades of *C. godetiae*, *C. fioriniae* or *C. nymphaeae*; that are already reported to possess high genetic diversity [12]. Thus, an additional phylogenetic analysis was performed incorporating more sequences from non-type strains of *C. godetiae, C. fioriniae* and *C. nymphaeae.* The multigene sequence alignment contained 2159 characters [ITS: 1–551; *act*: 552–822; *chs1*: 823–1096; *gapdh*: 1097–1361; *his3*: 1362–1748; *tub2*: 1749–2159], including gaps. Fifty ambiguous characters were excluded from the alignment and of the remaining 2109 included characters: 1564 characters were constant; 300 variable characters were parsimony-uninformative and 245 characters were parsimony-informative. The parsimony analysis yielded 205 equally most parsimonious trees, the topology of one of which is shown in Figure 2 [tree length (TL) = 703, consistency index (CI) = 0.869, retention index (RI) = 0.984, rescaled consistency index (RC) = 0.855, homoplasy index (HI) = 0.131]. Based on the Bayesian, MP and ML phylogenetic analyses (Figure 2), the isolates from almond were identified as *C. godetiae* and the isolates from anemone and strawberry were identified as *C. nymphaeae*. The NCBI-BLAST percent sequence identity of *C. godetiae* and *C. nymphaeae* with their closely related taxa is mentioned in the Supplementary Table S1. The number of parsimony-informative characters for each gene dataset is described in Supplementary Table S2.

### 2.4. Phylogenetic Analyses of Isolates Belonging to the C. boninense Species Complex

**Figure 3:**
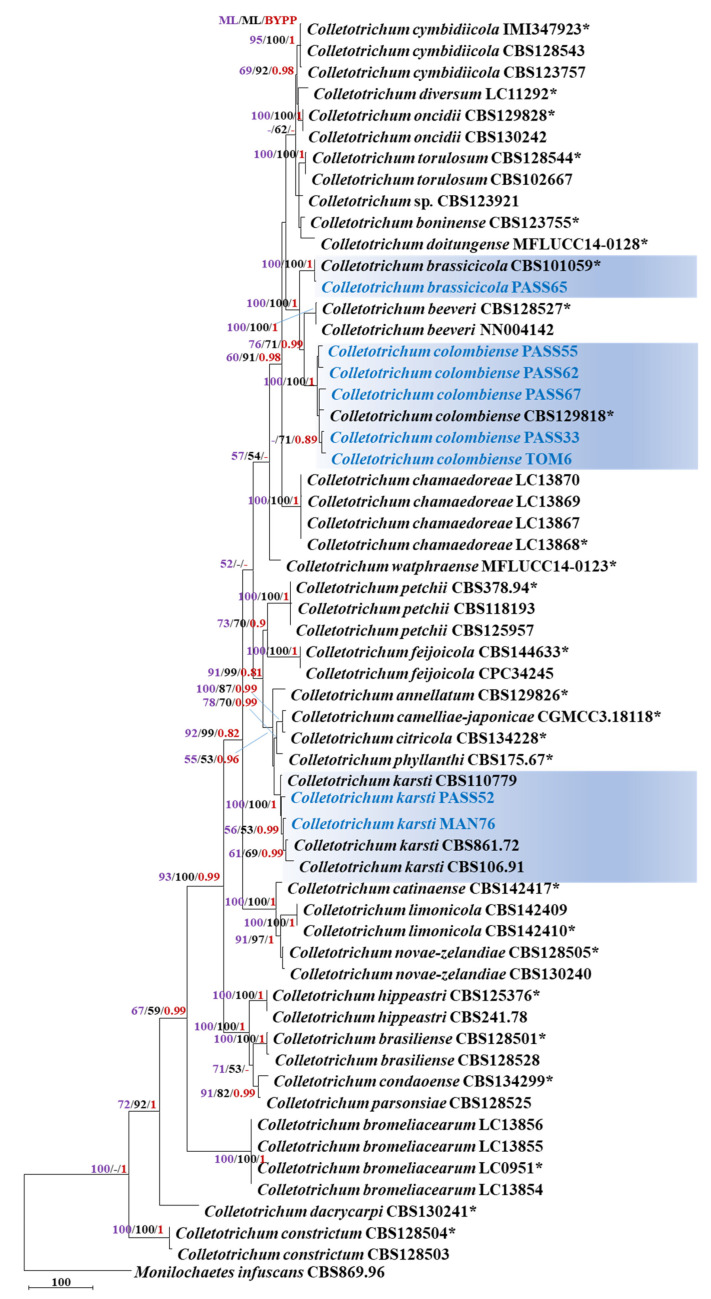
First of 63 equally most parsimonious trees obtained from a heuristic search of the combined ITS, *act*, *chs1*, *gapdh*, *his3* and *tub2* sequence alignment of the *Colletotrichum* isolates in the Boninense complex. The MP and ML bootstrap support values (>50%) and Bayesian posterior probabilities (>0.90) are displayed at the nodes (MP/ML/BYPP). The tree was rooted to *Monilochaetes infuscans* (CBS 869.96). The bar indicates 100 changes. The isolates from this study are highlighted in blue. (* = Type strain).

A concatenated dataset of six genes (ITS, *act*, *chs1*, *gapdh*, *his3* and *tub2*) was used for phylogenetic analyses of eight *C. boninense* species complex members in this study. *Monilochaetes infuscans* (CBS 869.96) was used as the outgroup, and 50 species in the Boninense complex were used as references in the analyses. The multigene sequence alignment contained 2246 characters [ITS: 1–569; *act*: 570–849; *chs1*: 850–1126; *gapdh*: 1127–1427; *his3*: 1428–1821; *tub2*: 1822–2246], including gaps. Eighty-one ambiguous characters were excluded from the alignment and of the remaining 2165 included characters: 1383 characters were constant; 216 variable characters were parsimony-uninformative and 566 characters were parsimony-informative. The parsimony analysis yielded 63 equally most parsimonious trees, the topology of one of which is shown in Figure 3 [tree length (TL) = 1545, consistency index (CI) = 0.674, retention index (RI) = 0.870, rescaled consistency index (RC) = 0.586, homoplasy index (HI) = 0.326]. The Bayesian analysis of the combined alignment, lasted 5,000,000 generations, and resulting trees were used to calculate the posterior probabilities. Bootstrap support values of the MP analysis (MP > 50%), ML analysis (ML > 50%) and the BI posterior probabilities (PP > 0.90) are depicted at the branch nodes (Figure 3). 

The overall branch support for the observed tree was high. Based on the MP and ML phylogenetic analyses, PASS33, PASS55, PASS62, PASS67 (passiflora) and TOM6 (tamarilla) isolates clustered with the ex-type sequences of *C. colombiense* CBS129818 (MP 100%, ML 100%, PP 1.0); PASS65 (passiflora) clustered with the ex-type sequences of *C. brassicicola* CBS101059 (MP 100%, ML 100%, PP 1.0); MAN 76 (mango) and PASS52 (passiflora) clustered with the sequences of *C. karsti* (MP 100%, ML 100%, PP 1.0). These isolates were also recovered as monophyletic with strong bootstrap support in four out of six individual gene trees (*act*, *chs1, his3* and *tub2*) (data not shown). The phylogenetic analyses of the Boninense complex identified isolates of *C. brassicicola*, *C. colombiense* and *C. karsti* as pathogenic to non-host strawberry fruits.

### 2.5. Pathogenicity Testing

The pathogenicity of all the *Colletotrichum* isolates used in this study from their original host of isolation were already studied and published elsewhere [20,21,23]. In this study, we assessed the cross-inoculation potential of the *Colletotrichum* isolates to non-hosts. Strawberry was used as an experimental non-host, and symptoms were verified for the different representative *Colletotrichum* isolates (*C. godetiae*—ALM-KSH10, ALM-BZR82; *C. nymphaeae*—TUT-5954, STR-101, ANE-NL4, ANE-HV83C; *C. tamarilloi*—TOM-10, *C. acutatum*—ANE-NL12, *C. fioriniae*—APL2, ALM-US4; *C. karsti*—MAN76, PASS33; *C. colombiense*—TOM6, *C. brassicicola*—PASS65, *C. sydowii*—PASS35, *C*. *gloeosporioides s. l.*—Cg-Sc-A1, APL7). The *C*. *gloeosporioides s. l.* isolate Litchi-Cg2 could not be assessed for pathogenicity due to inadequate production of conidia.

**Figure 4:**
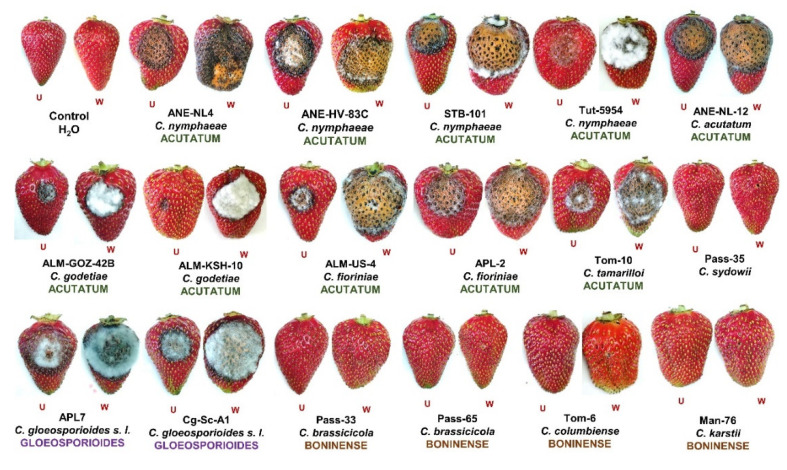
Anthracnose lesions on detached unwounded (UW) and wounded (W) strawberry fruits 7 days after inoculation for each representative isolate and species, within each *Colletotrichum* species complex used in this study.

**Figure 5:**
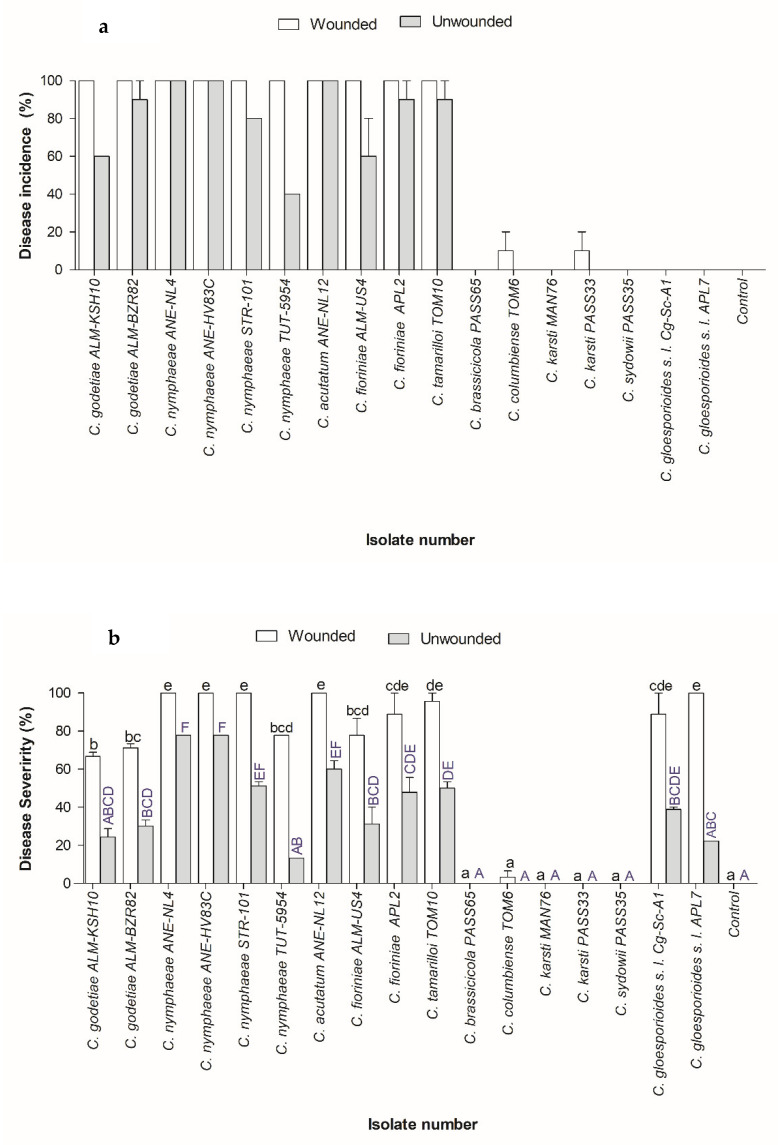
Bar diagram showing the (**a**) percent disease incidence and (**b**) percent disease severity of the representative *Colletotrichum* isolates used in this study. White bars = wounded fruits; gray bars = unwounded fruits. Data presented are the mean ± standard error. Means with different superscript letters are significant according to Tukey’s post-hoc test (*p* < 0.05).

The *Colletotrichum* species belonging to the Acutatum and Gloeosporioides species complexes exhibited anthracnose symptoms on strawberry fruits after 7 days of inoculation, whereas isolates belonging to the Boninense species complex and *C. sydowii* did not exhibit any noticeable anthracnose disease symptoms on inoculated fruit. Particularly, *C. nymphaeae* is an aggressive pathogen of strawberry in Israel, exhibiting 100% disease incidence and 75–100% disease severity in wounded inoculations. In unwounded inoculations, the disease incidence remained low. However, *C. nymphaeae* caused increased disease incidence and severity in unwounded inoculations as well. Interestingly, the *C. acutatum sensu stricto* and *C. nymphaeae* isolates from anemone were able to cross-infect strawberry. Similarly, *C. godetiae* and *C. fioriniae* isolates from almond, *C. fioriniae* isolate from apple and *C. tamarilloi* isolates from tamarillo were capable of infecting strawberry under wounded, as well as unwounded, conditions. This implies that the members of the Acutatum and Gloeosporioides species complexes used in this study are key pathogens of strawberry fruits and possess the potential for cross-infection. Detailed results of the pathogenicity tests are presented in Figure 4 and Figure 5 and in Supplementary Tables S3 and S4.

## 3. Discussion

### 3.1. Molecular Phylogeny of Isolates of Colletotrichum

Over the past decade, the phylogeny of the genus *Colletotrichum* has been in flux [24]. Prior to that, ITS sequencing was used to delineate isolates to the major “species”, e.g., *C. gloeosporioides*, *C. acutatum*, *C. coccodes*, etc. [24]. Thus, the single-gene phylogeny based on ITS sequencing did not result in reliable species diagnostics and identification [25]. However, since 2012, the main species clusters have been redefined according to multi-locus sequencing. For example, the Acutatum species complex has been defined according to phylogenetic analyses of the ITS, *act*, *tub2*, *chs1*, *gapdh* and *his3* genes [12]; the Gloeosporioides species complex has been defined according to analyses of up to eight genes [18]; and the Boninense species complex has been defined according to phylogenetic analyses of the ITS, *act*, *tub2*, *chs1*, *gapdh*, *his3* and *cal* genes [13]. Thus, the multi-locus sequencing of many populations of *Colletotrichum* has resulted in the renaming of undescribed cryptic species within the systematics of the genus. To date, a comprehensive systematics of the genus *Colletotrichum* includes a list of 16 species complexes, while an additional 15 novel single-isolate representatives were also described [26].

### 3.2. Previous Characterization of Strains

Past studies have characterized the current *Colletotrichum* isolate collection from Israel and elsewhere to species based on various molecular techniques. For example, isolates of *C. gloeosporioides* from avocado and other hosts were characterized according to AP-PCR, A+T-rich mitochondrial DNA, nuclear DNA and rDNA analyses; however, none of these methods were suitable in differentiating between the species from this complex [20]. Similarly, in additional studies, the *C. acutatum* isolates from various hosts, including anemone, olive and strawberry, were characterized to the specific complex using ITS sequencing, however, under limited conditions that did not coincide with pathogenicity tests [22]. Likewise, isolates within the species complex, Boninense, were originally identified based on ITS and rDNA sequencing [27]. In all of these past studies, the ITS sequence alone was not informative enough and did not delineate isolates to the species level within each *Colletotrichum* complex [20,21,22].

### 3.3. Colletotrichum of Almond

Under the circumstances, *Colletotrichum* affecting almond appears to be diverse; however, among the studied cases, the different populations were clustered within the Acutatum species complex. In Israel, the population of *Colletotrichum* is specific to almond and appears to be clonal and unique; however, ITS sequencing alone was not able to differentiate between the population from the US and those affecting almond from Australia [20]. The Australian populations were distinct from those found in Israel, however, and were more closely related to those affecting almond from the US [28].

As far as pathogenicity is concerned, all of the above populations of *Colletotrichum* affecting almond were pathogenic to the crop. In Israel, the specificity was demonstrated as follows: isolates that originated from almond were unique, and none of these isolates were detected within the populations affecting avocado, characterized within the Gloeosporioides complex; the anthracnose-affected almond orchards were cultivated adjacent, at a 5–10 m distance, to the affected avocado orchards [21]. Therefore, there is no need to be concerned that cross-inoculation from almond to avocado and vice versa takes place in the field. Thus, management protocols for disease reduction in each crop can be developed individually without any threat of cross-infection taking place between both of these crops.

### 3.4. Colletotrichum Affecting Anemone and Strawberry

Another case study in Israel, concerns the molecular identity of populations of *Colletotrichum* that affect anemone and strawberry. Anthracnose disease of anemone and strawberry is common, and both crops can be affected annually in Israel [21]. In certain instances, the same farmer can cultivate both crops in close proximity, when the infection takes place simultaneously. In the past, populations of the Acutatum complex were reported as causal agents of both crops, and it was shown that cross-inoculation of certain isolates/populations takes place under controlled conditions [21] and under field conditions (Freeman, pers. comm.).

### 3.5. Pathogenicity

The pathogenicity of *Colletotrichum* producing anthracnose symptoms can be evaluated on detached or attached plant structures. It has been shown, on the one hand, that detached fruit are not ideal “substrates” for pathogenicity testing; however, on the other hand, post-harvest evaluations are conducted primarily on detached fruit, e.g., pathogenicity and virulence on avocado [29]. In this study, the artificial inoculation of *Colletotrichum* isolates delineated to specific species within each complex were artificially inoculated on strawberry fruit. Reliable results were achieved that differentiated between specific species, indicating that this method is accurate in evaluating the pathogenic and virulence specificity for the isolates tested in this study.

Since the members of the Acutatum and Gloeosporioides species complexes are known pathogens of strawberry, all of the tested representative isolates belonging to these two species complexes were able to cross-infect strawberry fruits in the wounded as well as unwounded inoculations. However, slight differences in disease symptoms and severity were observed among the different isolates, such as those from anemone (ANE-NL4) and strawberry (TUT-5954) that may be attributed to the high genetic diversity within *C. nymphaeae*. Similar to the present study, *C. acutatum* and *C. fioriniae* have been previously reported to be associated with strawberry anthracnose symptoms. This is the first report of *C. tamarilloi* causing strawberry anthracnose. 

The *Colletotrichum* isolates belonging to the Boninense species complex did not cause anthracnose symptoms in wounded or unwounded inoculations. Thus far, there is one report of the association of *C. boninense* and *C. karsti* with strawberry in China [30,31]. However, *C. brassicicola, C. colombiense* and *C*. *sydowii* have not been previously associated with strawberry [32], and, as in this study, *C*. *sydowii* was found to be non-pathogenic on detached strawberry fruits. Thus, according to the pathogenicity assays reported in this study, *Colletotrichum* isolates possess cross-infection potential within their respective species complex.

### 3.6. Colletotrichum Species Affecting Anemone, Strawberry and Almond

Within the Acutatum species complex, *C. acutatum*, *C. fioriniae*, *C. godetiae*, *C*. *nymphaeae* and *C*. *simmondsii* have been reported as pathogens of almond and strawberry fruit, while *C. acutatum*, *C. fioriniae* and *C*. *nymphaeae* have been reported on anemone [32]. In this study, a polyphasic approach of multigene phylogenetic analysis, morphological characterization and pathogenicity assays was used to characterize 36 *Colletotrichum* isolates that were previously identified based on AP-PCR and ITS sequence data alone. Similar to previous studies, *C. godetiae* was associated with almonds, and *C*. *nymphaeae* was associated with anemone and strawberry in Israel. The genealogical concordance was also evaluated using individual gene trees; however, there is a need to improve the phylogenetically informative barcode markers for the Acutatum species complex. The percent parsimony-informative characters for the various genes used for the phylogenetic analysis of the Acutatum species complex is quite low (Supplementary Table S2). In the case of the Gloeosporioides species complex, intergenic regions of the *apn2* and *MAT1-2-1* gene (ApMat) markers, along with the glutamine synthase (gs) gene, provide an efficient species-level resolution [33,34].

## 4. Materials and Methods

### 4.1. Fungal Isolates, Growth Conditions and Morphological Characterization

The monoconidial *Colletotrichum* isolates used in this study are listed in Table 2. The *Colletotrichum* isolates associated with almond, strawberry and anemone were isolated between 1993 and 1995, in Israel [20,21,22]. The isolates associated with passiflora, tamarillo and mango were isolated between 1998 to 2000, in Colombia [27]. The isolates associated with apple, pecan and peach originated in the US [22]. The *Colletotrichum* isolates were retrieved from the −80 °C facility of the Freeman laboratory at the ARO, revived on potato dextrose agar (PDA, Difco, USA) plates and grown at 25 °C for morphological characterization (colony morphology and growth rate). To increase sporulation, the isolates were grown on modified Mathur’s MS semi-selective (M3S) agar medium (per liter composition: 2.5 gm of MgSO_4_·7H_2_O, 2.7 gm of KH_2_PO_4_, 1 gm of peptone, 1 gm of yeast extract, 10 gm of sucrose and 20 gm of agar) [29] and synthetic nutrient agar (SNA) medium (per liter composition: 1 gm of KH_2_PO_4_, 1 gm of KNO_3_, 0.5 gm of MgSO_4_·7H_2_O, 0.5 gm of KCl, 0.2 gm of glucose, 0.2 gm of sucrose and 20 gm of agar) [35]. Microscopic slides were prepared in 1% lactic acid or water. For each representative isolate, the shape and size of the conidia and conidiogenous cells were measured using bright field microscopy (Nikon, Japan) and measured via NIS-Elements image analysis software (Nikon). At least 50 measurements were made for the length and width of the conidia. The growth rate was determined by measuring the colony diameter after 7 days (mm/day). Morphological characteristics are listed in Table 1.

### 4.2. DNA Extraction, PCR Amplification and Sequencing

The 36 representative *Colletotrichum* isolates used in this study were grown at 25 °C in 20 mL broth of glucose minimal media [29], without shaking. The fungal mycelia were harvested after 7 days by vacuum filtration. DNA was extracted according to Freeman et al. [36]. The partial sequence of actin (*act*), chitin synthase (*chs1*), glyceraldehyde-3-phosphate dehydrogenase (*gapdh*), histone (*his3*), β-tubulin (*tub2*) and the internal transcribed spacer (ITS) region were amplified and sequenced using the primer pairs ACT-512F+ACT-783R [37], Bt-2A + Bt-2B [38], CHS-79F+CHS-354R [37], GDF1+GDR1 [39], CYLH3F+CYLH3R [40] and ITS-5+ITS-4 [41], respectively. The PCR reactions were carried out in a 20 µL volume. Each reaction tube contained 1.5 µL of total genomic DNA (100 ng/µL concentration), 10 µL of 2× PCRBIO HS Taq Mix Red (PCR Biosystems, www.pcrbio.com, accessed on 27 June 2022), 0.5 µL each of 10 µM forward and reverse primer and 7.5 µL of sterile water in a thermocycler (Biometra TAdvanced, Analytik-Jena, Jena, Germany). The cycling parameters were: initial denaturation at 95 °C for 4 min, followed by 34 cycles of denaturation at 95 °C for 30 s, annealing for 30 s (at 58 °C for *act*, 56 °C for *chs1*, 60 °C for *gapdh*, 52 °C for *his3*, 55 °C for *tub2* and 54 °C for ITS), extension at 72 °C for 45 s and a final extension at 72 °C for 7 min. The PCR products were separated by 1.2% agarose gel electrophoresis, purified with the NucleoSpin Gel and PCR Clean-up kit (Macherey-Nagel, Dueren, Germany) and quantified using a Nanodrop Spectrophotometer ND-1000 (Thermo Fisher Scientific, Wilmington, NC, USA). Both strands of the purified PCR products were sequenced at Macrogen Europe (http://www.macrogen.com, accessed on 27 June 2022). The sequences generated in this study were submitted to NCBI for GenBank accession numbers (Table 2). 

### 4.3. Phylogenetic Analyses

The forward and reverse sequences for each gene were checked for quality and assembled using MEGA-X v. 10.1.17 [42]. The different gene regions were concatenated using SequenceMatrix v. 1.7.8 [43]. Ambiguous regions from the multiple sequence alignment were not included in the analyses, and the gaps were considered missing data (N). The six-gene dataset (*act*, *cal*, *gapdh*, *his3*, ITS and *tub2*) was used for the phylogenetic analyses of the *Colletotrichum* isolates belonging to the *C. acutatum* and *C. boninense* species complex. For the remaining three isolates belonging to the *C. gloeosporioides* species complex, the phylogenetic analyses comprised a five-gene dataset (*act*, *chs1*, *gapdh*, ITS and *tub2*). The reference type–strain sequences for the analyses were retrieved from GenBank [22] and are listed in Supplementary Tables S5–S8.

Phylogenetic analyses were conducted using a maximum parsimony (MP), maximum likelihood (ML) and Bayesian inference (BI) methods. The maximum parsimony analysis was conducted using PAUP v. 4.0b10 [44], as detailed earlier in Sharma et al. [29]. The maximum likelihood phylogeny was inferred using RAxML-HPC2 under the GTR-GAMMA model in the CIPRES portal [45,46], and the branch support was evaluated by bootstrap analysis of 100 replicates (-m GTRGAMMA -p 12345 -f a -N 100 -x 12345 --asc-corr lewis). The Bayesian inference of the phylogeny was estimated using MrBayes version 3.2.7a [47] in the CIPRES portal with four MCMC chains of 5,000,000 generations. The sample trees were recorded every 10^3^ generations, and 25% of the initial trees were discarded as burn-in. For each gene dataset, a suitable model for the estimation of phylogeny was evaluated, and ML analysis was carried out using MEGA-X and the “one click mode” tree analysis method, available at www.phylogeny.fr, accessed on 27 June 2022 [48] (data not shown). The resulting trees from each analysis were viewed in FigTree v. 1.4.4 [49] and edited in Microsoft PowerPoint 2016. 

### 4.4. Pathogenicity Assay

The pathogenic potential of the representative isolates was evaluated by artificial inoculations of unripe strawberry fruit (light green to light red in color), cv. Peles, which is susceptible to anthracnose, that originated from a disease-free organic strawberry farm in the Sharon area of Israel. Prior to inoculation, the calyx and peduncle were trimmed, and the fruits were washed under running water. The conidia were harvested as described [29] and adjusted to 2 × 10^7^ conidia/mL. After air drying, the fruits were inoculated with 10 µL of conidial solution at wounded (pin-pricked) and unwounded sites. In the control fruits, sterile saline solution was used for inoculation. The inoculated fruits were maintained in a moist chamber at room temperature (25–27 °C) and observed daily for the appearance of anthracnose symptoms. The disease severity and disease incidence were calculated as described in Sharma et al. [50] and detailed in Supplementary Table S3. The disease score was recorded and indicated in Supplementary Table S4. The statistical significance for the disease severity was calculated according to Tukey’s post-hoc test (*p* < 0.05) (Figure 5b).

## 5. Conclusions

ITS sequence data alone is not useful for identification and differentiation of species from the genus *Colletotrichum*. The derived classification is inaccurate and groups the isolates to a species complex but does not delineate them to species accurately. Currently, multi-locus sequence data has been adopted and is routinely used for delineating and accurately describing members of the *Colletotrichum* genus to new species within each complex. Additionally, the cross-infection potential of different *Colletotrichum* species was assessed, which provided insights into their diverse host-range and pathogenic potential on non-hosts. Plant breeders should consider the broad host range and high genetic diversity of the *Colletotrichum* species when developing disease resistant cultivars.

## Figures and Tables

**Table 1:** Colony morphology and growth rate on PDA and conidial characteristics of the representative *Colletotrichum* species from this study.

Isolate	Complex	Colony Morphology	Conidia Length (μm)	Conidia Width (μm)	Growth Rate (mm/Day)
*C.godetiae* ALM-KSH10	Acutatum	cottony, grey aerial mycelium; reverse dark grey to pale orange in center	(9.6–18.9) mean = 13.81 ± 0.24	(3.5–5.7) Mean = 4.68 ± 0.06	5.4
*C.godetiae* ALM-BZR82	Acutatum	cottony, grey aerial mycelium; reverse dark grey to pale orange in center	(8.9–19.3) mean = 13.52 ± 0.46	(2.4–5.6) Mean = 4.26 ± 0.15	5.3
*C.nymphaeae* ANE-NL4	Acutatum	cottony, white aerial mycelium; reverse pale yellow to pale orange in center, with rings	(7.9–16.7) mean = 13.84 ± 0.25	(2.7–4.7) Mean = 3.88 ± 0.07	7.0
*C.nymphaeae* ANE-HV83C	Acutatum	white colony with orange center, reverse pale white to orange	(11.5–21.2) mean = 14.17 ± 0.20	(2.6–5.5) Mean = 4.30 ± 0.08	6.9
*C.nymphaeae* STR-101	Acutatum	cottony, white aerial mycelium; reverse pale yellow to pale orange in center, with rings	(9.3–16.5) mean = 12.82 ± 0.18	(2.8–4.7) Mean = 3.64 ± 0.05	6.8
*C.nymphaeae* TUT-5954	Acutatum	cottony, white aerial mycelium, reverse pale white with orange center	(7.6–15.7) mean = 12.07 ± 0.21	(2.7–4.7) Mean = 3.74 ± 0.06	7.2
*C. acutatum* ANE-NL12	Acutatum	cottony, white aerial mycelium, reverse pale white with dark red center	(9.5–16.4) mean = 13.49 ± 0.20	(3.4–5.8) Mean = 4.62 ± 0.06	7.3
*C. fioriniae* ALM-US4	Acutatum	white to light pink with greenish gray center with conidial mass; reverse whitish to light pink in the center	(7.0–13.2) mean = 10.69 ± 0.20	(3.1–5.9) Mean = 4.45 ± 0.07	7.7
*C. fioriniae* APL2	Acutatum	pinkish red colony with greenish gray center, reverse light pink with dark pink rings	(7.1–14.3) mean = 10.27 ± 0.22	(2.5–4.9) Mean = 3.74 ± 0.07	7.7
*C. tamarilloi* TOM10	Boninense	white colony with orange center, reverse pale white to orange	(8.1–16.3) mean = 11.44 ± 0.25	(2.9–5.2) Mean = 3.79 ± 0.07	6.1
*C. brassicicola* PASS65	Boninense	white with orange and black conidiomata in the center and pale orange center	(12.6–18.5) mean = 15.58 ± 0.15	(4.3–7.8) Mean = 5.74 ± 0.08	3.1
*C. columbiense* TOM6	Boninense	greyish black mycelia with white margins, reverse grey	(13.1–17.5) mean = 14.98 ± 0.14	(4.8–8.3) Mean = 6.56 ± 0.10	7.3
*C. columbiense* PASS33	Boninense	cottony, grey aerial mycelium with rings; reverse white with grey-orange center	(11.5–17.2) mean = 14.66 ± 0.16	(4.8–7.4) Mean = 5.90 ± 0.09	8.3
*C. karsti* MAN76	Boninense	cottony, white mycelium with grey rings of conidiomata, reverse pale white	(10.5–18.9) mean = 15.20 ± 0.29	(3.6–8.5) Mean = 6.02 ± 0.15	7.1
*C. sydowii* PASS35	Sydowii	greyish black mycelia with white margins, reverse dark grey	(14.8–22.5) mean = 18.74 ± 0.38	(4.7–8.5) Mean = 6.36 ± 0.17	6.8
*C. gloesporioides s. l.* Cg-Sc-A1	Gloeosporioides	cottony, white aerial mycelium with grey center, reverse pale yellow with dark grey center	(9.6–19.0) mean = 16.5 ± 0.22	(4.0–6.6) Mean = 5.17 ± 0.07	11.4
*C. gloesporioides s. l.* APL7	Gloeosporioides	cottony, white aerial mycelium with grey center, reverse pale yellow	(9.6–15.9) mean = 13.19 ± 0.16	(3.8–7.0) Mean = 5.30 ± 0.09	9.4

**Table 2:** Details of host, country of origin and species complex of *Colletotrichum* isolates used in the phylogenetic analyses along with the GenBank accession numbers of the sequences (N.S. = not sequenced).

Isolate	Complex	Taxon	Country	Host	ITS	*act*	*chs1*	*gapdh*	*his3*	*tub2*
ANE-NL-12	Acutatum	*Colletotrichum acutatum*	Netherlands	Anemone	ON631980	ON707522	ON707486	ON707635	ON707568	ON707601
ALM-US-4	Acutatum	*Colletotrichum fioriniae*	Israel	Almond	ON631977	ON707519	ON707483	ON707632	ON707565	ON707598
APL-2	Acutatum	*Colletotrichum fioriniae*	USA	Apple	ON631983	ON707525	ON707489	ON707638	ON707571	ON707604
PCH-8	Acutatum	*Colletotrichum fioriniae*	USA	Peach	ON631995	ON707537	ON707501	ON707650	ON707580	ON707614
PCN-5	Acutatum	*Colletotrichum fioriniae*	USA	Pecan	ON631996	ON707538	ON707502	ON707651	ON707581	ON707615
ALM-BZR-82 = CBS 149276	Acutatum	*Colletotrichum godetiae*	Israel	Almond	ON631968	ON707510	ON707474	ON707623	ON707556	ON707589
ALM-GOZ-36E	Acutatum	*Colletotrichum godetiae*	Israel	Almond	ON631969	ON707511	ON707475	ON707624	ON707557	ON707590
ALM-GOZ-42B	Acutatum	*Colletotrichum godetiae*	Israel	Almond	ON631970	ON707512	ON707476	ON707625	ON707558	ON707591
ALM-GVA-6A	Acutatum	*Colletotrichum godetiae*	Israel	Almond	ON631971	ON707513	ON707477	ON707626	ON707559	ON707592
ALM-GZT-1F	Acutatum	*Colletotrichum godetiae*	Israel	Almond	ON631972	ON707514	ON707478	ON707627	ON707560	ON707593
ALM-KN-17	Acutatum	*Colletotrichum godetiae*	Israel	Almond	ON631973	ON707515	ON707479	ON707628	ON707561	ON707594
ALM-KSH-10 = CBS 149275	Acutatum	*Colletotrichum godetiae*	Israel	Almond	ON631974	ON707516	ON707480	ON707629	ON707562	ON707595
ALM-KYZ-6W	Acutatum	*Colletotrichum godetiae*	Israel	Almond	ON631975	ON707517	ON707481	ON707630	ON707563	ON707596
ALM-NRB-30K	Acutatum	*Colletotrichum godetiae*	Israel	Almond	ON631976	ON707518	ON707482	ON707631	ON707564	ON707597
STR-101 = CBS 149278	Acutatum	*Colletotrichum nymphaeae*	Israel	Strawberry	ON631997	ON707539	ON707503	ON707652	ON707582	ON707616
STR-3	Acutatum	*Colletotrichum nymphaeae*	Israel	Strawberry	ON631998	ON707540	ON707504	ON707653	ON707583	ON707617
TUT-137	Acutatum	*Colletotrichum nymphaeae*	Israel	Strawberry	ON632001	ON707543	ON707507	ON707656	ON707586	ON707620
TUT-149	Acutatum	*Colletotrichum nymphaeae*	Israel	Strawberry	ON632002	ON707544	ON707508	ON707657	ON707587	ON707621
TUT-5954 = CBS 149277	Acutatum	*Colletotrichum nymphaeae*	Israel	Strawberry	ON632003	ON707545	ON707509	ON707658	ON707588	ON707622
ANE-3A = CBS 149280	Acutatum	*Colletotrichum nymphaeae*	Israel	Anemone	ON631978	ON707520	ON707484	ON707633	ON707566	ON707599
ANE-HV-83C = CBS 149279	Acutatum	*Colletotrichum nymphaeae*	Israel	Anemone	ON631979	ON707521	ON707485	ON707634	ON707567	ON707600
ANE-NL4	Acutatum	*Colletotrichum nymphaeae*	Netherlands	Anemone	ON631981	ON707523	ON707487	ON707636	ON707569	ON707602
ANE-UK-31	Acutatum	*Colletotrichum nymphaeae*	United Kingdom	Anemone	ON631982	ON707524	ON707488	ON707637	ON707570	ON707603
TOM-10	Acutatum	*Colletotrichum tamarilloi*	Colombia	Tamarillo	ON631999	ON707541	ON707505	ON707654	ON707584	ON707618
PASS-65	Boninense	*Colletotrichum brassicicola*	Colombia	Passiflora	ON631993	ON707535	ON707499	ON707648	ON707578	ON707612
PASS-33	Boninense	*Colletotrichum colombiense*	Colombia	Passiflora	ON631988	ON707530	ON707494	ON707643	ON707573	ON707608
PASS-55	Boninense	*Colletotrichum colombiense*	Colombia	Passiflora	ON631991	ON707533	ON707497	ON707646	ON707576	ON707610
PASS-62	Boninense	*Colletotrichum colombiense*	Colombia	Passiflora	ON631992	ON707534	ON707498	ON707647	ON707577	ON707611
PASS-67	Boninense	*Colletotrichum colombiense*	Colombia	Passiflora	ON631994	ON707536	ON707500	ON707649	ON707579	ON707613
TOM-6	Boninense	*Colletotrichum colombiense*	Colombia	Tamarillo	ON632000	ON707542	ON707506	ON707655	ON707585	ON707619
MAN-76	Boninense	*Colletotrichum karsti*	Colombia	Mango	ON631987	ON707529	ON707493	ON707642	ON707572	ON707607
PASS-52	Boninense	*Colletotrichum karsti*	Colombia	Passiflora	ON631990	ON707532	ON707496	ON707645	ON707575	ON707609
PASS-35	Sydowii	*Colletotrichum sydowii*	Colombia	Passiflora	ON631989	ON707531	ON707495	ON707644	ON707574	N.S.
Litchi-Cg2	Gloeosporioides	*Colletotrichum aenigma*	Colombia	Litchi	ON631986	ON707528	ON707492	ON707641		N.S.
APL-7	Gloeosporioides	*Colletotrichum* sp.	USA	Aplle	ON631984	ON707526	ON707490	ON707639	N.S.	ON707605
CG-SC-A1	Gloeosporioides	*Colletotrichum* sp.	Israel	Salicornia	ON631985	ON707527	ON707491	ON707640	N.S.	ON707606

## Data Availability

The sequences generated in this study are deposited in the NCBI-GenBank.

## Supplementary Materials

The following supporting information can be downloaded at: https://www.mdpi.com/article/10.3390/plants11182373/s1.
