# Supplementary material for: Characterization of Colletotrichum Isolates from Strawberry and Other Hosts with Reference to Cross-Inoculation Potential
Source: Plants (Basel). 2022 Sep 11;11(18):2373. doi: 10.3390/plants11182373 (PMC9500779; doi:10.3390/plants11182373)
Supplement: Supplementary file 1 [file plants-11-02373-s001.zip › plants-1820980-supplementary.pdf]

Supplementary information

Characterization of *Colletotrichum* isolates from strawberry and other hosts with reference to cross-inoculation potential

Gunjan Sharma[1,2], Marcel Maymon[1], Vineet Meshram[1] and Stanley Freeman[1,*]

[1]Department of Plant Pathology and Weed Research, Institute of Plant Protection, Agricultural Research Organization, Volcani Institute, Rishon LeZion, 7505101, Israel.

[2]Present affiliation: Gujarat Biotechnology University, Gandhinagar, Gujarat, 382355, India.

ORCID (GS: 0000-0003-2199-9389; SF: 0000-0002-1904-2206)

*Corresponding author, E-mail: freeman@volcani.agri.gov.il

Phylogenetic analyses of isolates belonging to *C. gloeosporioides* species complex

For the phylogenetic analyses of three *C. gloeosporioides* species complex members from this study, a concatenated dataset of five genes (ITS, *gapdh*, *tub2*, *act*, and *chs-1*) was used. *Monilochaetes infuscans* (CBS 869.96) was used as the outgroup, and 56 species in the gloeosporioides complex were used as reference in the analysis. The multigene sequence alignment contained 1879 characters [ITS: 1-588; *act*: 589-870; *chs1*: 871-1144; *gapdh*: 1145-1428; *tub2*: 1429-1879], including gaps. Eighty-three ambiguous characters were excluded from the alignment and of the remaining 1796 included characters: 1137 characters were constant; 398 variable characters were parsimony-uninformative and 261 characters were parsimony-informative. The parsimony analysis yielded 7098 equally most parsimonious trees, the topology of one of which is shown in Supplementary Figure S1 [tree length (TL) = 1128, consistency index (CI) = 0.716, retention index (RI) = 0.821, rescaled consistency index (RC) = 0.588, homoplasy index (HI) = 0.284]. The Bayesian analysis of the combined alignment, lasted 5000000 generations, resulting in 10002 total trees of which 7502 trees were used to calculate the posterior probabilities. Bootstrap support values of the MP analysis (MP > 50%), and the BI posterior probabilities (PP > 0.90) are depicted at the branch nodes (Supplementary Figure S1). The overall branch support for the observed tree was poor. Based on the phylogenetic analyses, Litchi-Cg2 clustered with the ex-type sequences of *C. aenigma* ICMP18608 (MP 62%, PP 0.98); whereas APL7 and Litchi-Cg2 did not align with any strongly supported clades, thus we refer to these isolates in the manuscript as *C. gloeosporioides s. l.*

Supplementary Figure S1 First of 7098 equally most parsimonious trees obtained from a heuristic search of the combined ITS, *act*, *chs1*, *gapdh*, and *tub2* sequence alignment of the *Colletotrichum* isolates in the Gloeosporioides complex. The MP bootstrap support values (> 50%) and Bayesian posterior probabilities (> 0.80) are displayed at the nodes (MP/ML/BYPP).

The tree was rooted to *Monilochaetes infuscans* (CBS 869.96). The bar indicates 50 changes. The isolates from this study are highlighted in blue.

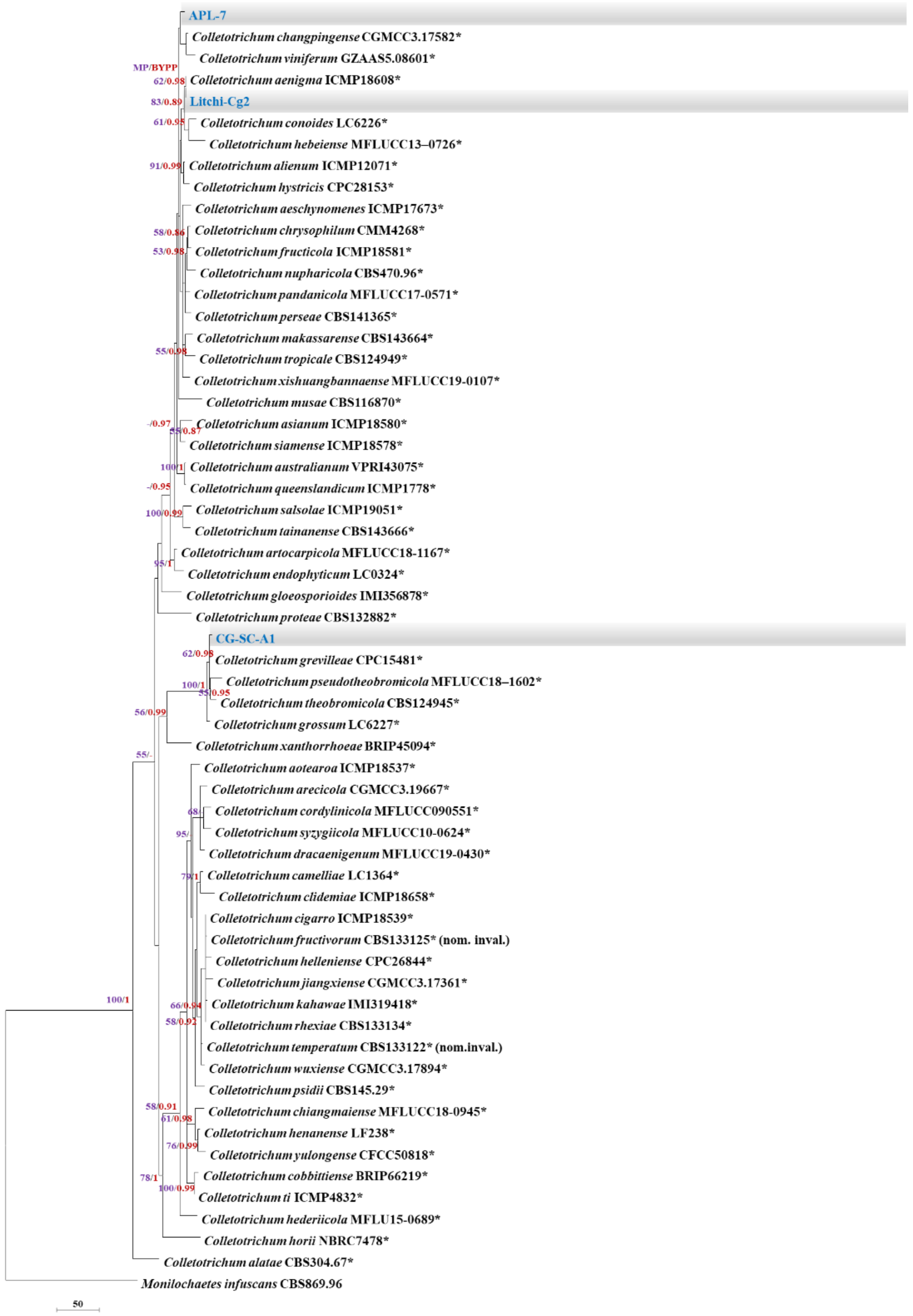

Supplementary Figure S2 *Colletotrichum godetiae* (a–f) Cultures grown on (a,c,e) PDA, (b,d,f) M3S, for 7 days at 25 °C. (g) conidia. (h) setae. (i) conidiophores and conidia. (j-p) appressoria. Scale bars: (g) = 20 μm, (h-p) = 10 μm. (Scale bar of p is applicable to j-o)

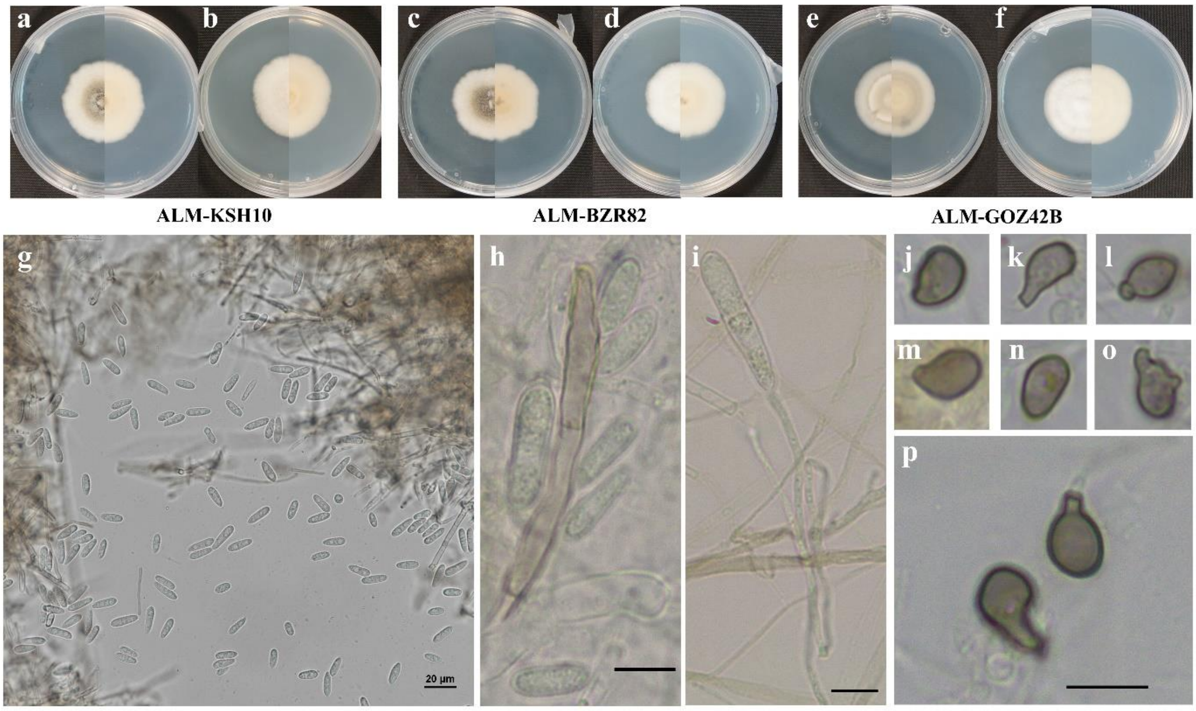

Supplementary Figure S3 *Colletotrichum nymphaeae* (a-l) Cultures on (a,c,e,g,i,k) PDA, (b,d,f,h,j,l) M3S after 7 days at 25 °C. (m,n) conidiophores and conidia. (o) conidia. Scale bars: (m-n) = 10 µm.

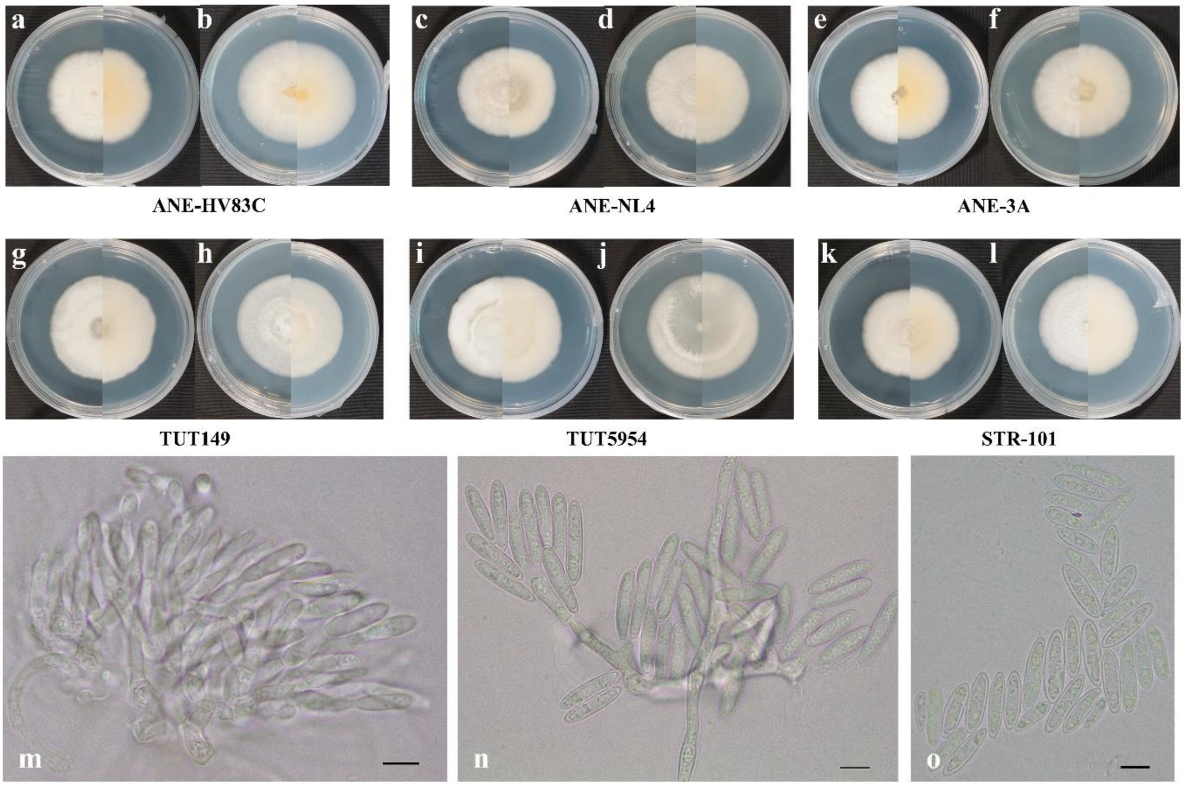

Supplementary Figure S4 Colony morphology (left, PDA, right M3S) and conidial features of the representative *Colletotrichum* species used in this study.

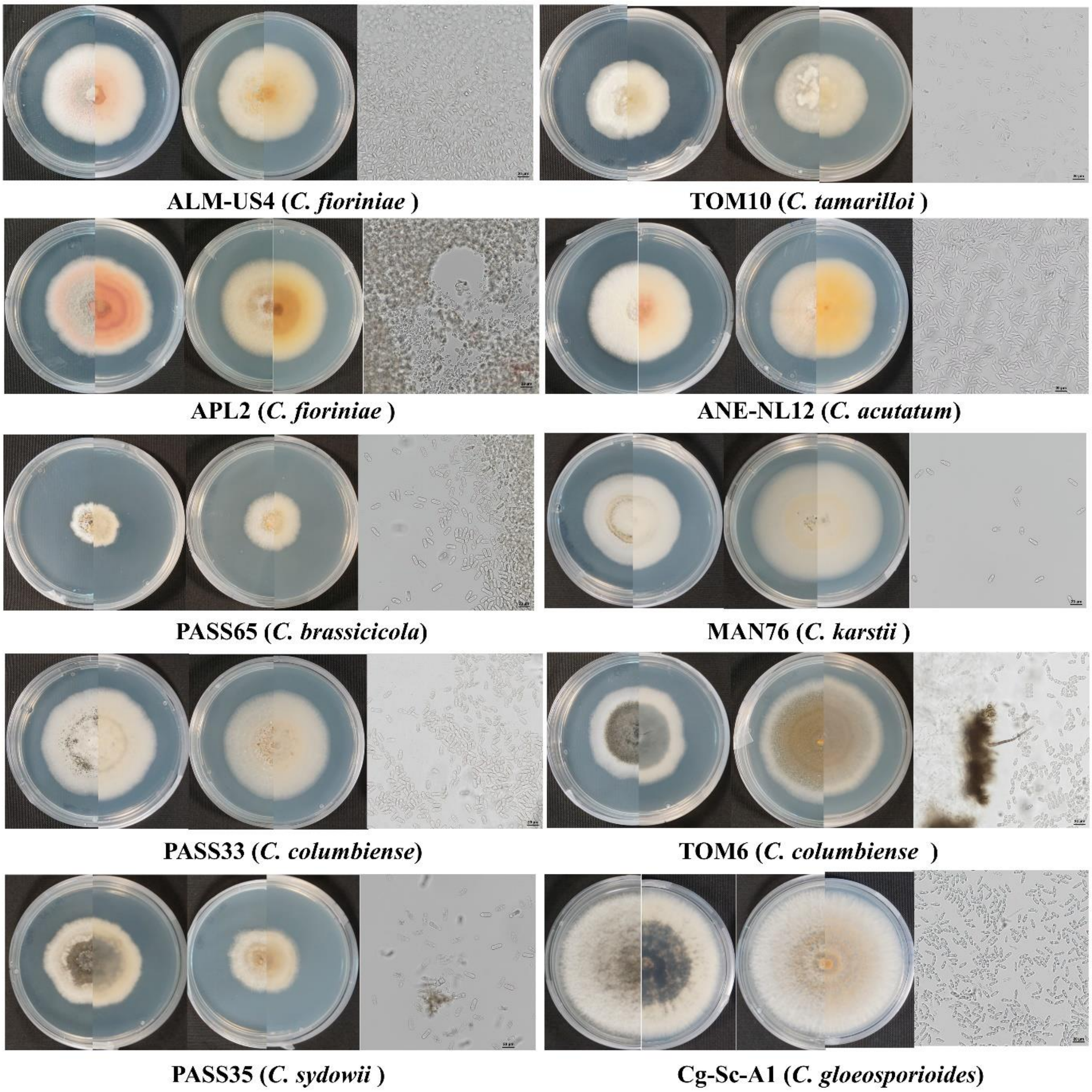

Supplementary Table S1 Results from the NCBI-BLAST search for the two representative isolates from almond and strawberry with their closely related taxa

	**Query = *C. godetiae* ALM-KSH10, Subject = *C. godetiae* CBS133.44***			**Query = *C. nymphaeae* STR101, Subject = *C. nymphaeae* CBS515.78***	
gene	Query coverage	% Identity	gene	Query coverage	% Identity
act	100%	99.19	*act*	100%	100
chs1	100%	99.63	*chs1*	100%	99.28
gapdh	100%	99.2	*gapdh*	100%	98.99
his3	100%	98.7	*his3*	100%	97.64
tub2	100%	100	*tub2*	100%	100
ITS	100%	99.81	ITS	100%	99.63

Supplementary Table S2 Number of parsimony informative character (PIC) in each gene dataset used in the phylogenetic analysis of the respective species complex (Acutatum, Boninense, Gloeosporioides)

Species complex	**Acutatum**			**Boninense**			**Gloeosporioides**		
**gene**	**PIC**	**No of characters in alignment**	**% PIC**	**PIC**	**No of characters in alignment**	**% PIC**	**PIC**	**No of characters in alignment**	**% PIC**
act	45	271	16.6	89	280	31.8	45	282	16.0
chs1	25	274	9.1	55	277	19.9	41	274	15.0
gapdh	48	265	18.1	142	301	47.2	88	284	31.0
his3	59	387	15.2	98	394	24.9	-	-	-
ITS	23	551	4.2	58	569	10.2	20	588	3.4
tub2	45	411	10.9	125	425	29.4	68	451	15.1

Supplementary Table S3 Percent disease incidence (PDI) and percent disease severity (PDS*) in strawberry fruits, 7 days post inoculation with the representative *Colletotrichum* isolates

Isolate	Control fruit	Replicate 1								Replicate 2								Mean			
		Test fruit 1	Test fruit 2	Test fruit 3	Test fruit 4	Test fruit 5	PDI	Σ(a+b)	PDS	Test fruit 6	Test fruit 7	Test fruit 8	Test fruit 9	Test fruit 10	PDI	Σ(a+b)	PDS	**PDI**	**SE (PDI)**	PDS	SE (PDS)
**WOUNDED**																					
C. dulcis sp. nov. ALM-KSH10	0	7	7	7	5	3	100	29	64.4	3	7	7	7	7	100	31	68.9	100	0	66.7	2.2
C. dulcis sp. nov ALM-BZR82	0	5	7	7	7	7	100	33	73.3	7	7	7	5	5	100	31	68.9	100	0	71.1	2.2
C. israelensis sp. nov. ANE-NL4	0	9	9	9	9	9	100	45	100.0	9	9	9	9	9	100	45	100.0	100	0	100.0	0.0
C. israelensis sp. nov. ANE-HV83C	0	9	9	9	9	9	100	45	100.0	9	9	9	9	9	100	45	100.0	100	0	100.0	0.0
C. israelensis sp. nov. STR-101	0	9	9	9	9	9	100	45	100.0	9	9	9	9	9	100	45	100.0	100	0	100.0	0.0
C. israelensis sp. nov. TUT-5954	0	7	7	7	5	9	100	35	77.8	7	7	7	5	9	100	35	77.8	100	0	77.8	0.0
C. acutatum ANE-NL12	0	9	9	9	9	9	100	45	100.0	9	9	9	9	9	100	45	100.0	100	0	100.0	0.0
C. fioriniae ALM-US4	0	3	9	9	5	5	100	31	68.9	9	9	9	5	7	100	39	86.7	100	0	77.8	8.9
C. fioriniae APL2	0	3	5	9	9	9	100	35	77.8	9	9	9	9	9	100	45	100.0	100	0	88.9	11.1
C. tamarilloi TOM10	0	9	9	9	9	9	100	45	100.0	9	9	9	7	7	100	41	91.1	100	0	95.6	4.4
C. brassicicola PASS65	0	0	0	0	0	0	0	0	0.0	0	0	0	0	0	0	0	0.0	0	0	0.0	0.0
C. columbiense TOM6	0	0	0	0	0	0	0	0	0.0	0	0	0	3	0	20	3	6.7	10	10	3.3	3.3
C. karsti MAN76	0	0	0	0	0	0	0	0	0.0	0	0	0	0	0	0	0	0.0	0	0	0.0	0.0
C. karsti PASS33	0	0	0	0	0	0	0	0	0.0	0	0	0	0	0	0	0	0.0	0	0	0.0	0.0
C. sydowii PASS35	0	0	0	0	0	0	0	0	0.0	0	0	0	0	0	0	0	0.0	0	0	0.0	0.0
C. gloesporioides s. l. Cg-Sc-A1	0	9	9	7	5	5	100	35	77.8	9	9	9	9	9	100	45	100.0	100	0	88.9	11.1
C. gloesporioides s. l. APL7	0	9	9	9	9	9	100	45	100.0	9	9	9	9	9	100	45	100.0	100	0	100.0	0.0

**UNWOUNDED**																					
C. dulcis sp. nov. ALM-KSH10	0	3	5	5	0	0	60	13	28.9	0	0	3	3	3	60	9	20.0	60	0	24.4	4.4
C. dulcis sp. nov ALM-BZR82	0	3	3	3	3	3	100	15	33.3	0	3	3	3	3	80	12	26.7	90	10	30.0	3.3
C. israelensis sp. nov. ANE-NL4	0	7	7	7	7	7	100	35	77.8	7	7	7	7	7	100	35	77.8	100	0	77.8	0.0
C. israelensis sp. nov. ANE-HV83C	0	7	7	7	7	7	100	35	77.8	7	7	7	7	7	100	35	77.8	100	0	77.8	0.0
C. israelensis sp. nov. STR-101	0	5	5	5	0	9	80	24	53.3	5	5	5	7	0	80	22	48.9	80	0	51.1	2.2
C. israelensis sp. nov. TUT-5954	0	0	0	0	3	3	40	6	13.3	0	0	0	3	3	40	6	13.3	40	0	13.3	0.0
C. acutatum ANE-NL12	0	5	5	7	7	5	100	29	64.4	5	5	5	5	5	100	25	55.6	100	0	60.0	4.4
C. fioriniae ALM-US4	0	5	5	5	3	0	80	18	40.0	0	0	0	5	5	40	10	22.2	60	20	31.1	8.9
C. fioriniae APL2	0	3	3	5	7	0	80	18	40.0	3	5	5	5	7	100	25	55.6	90	10	47.8	7.8
C. tamarilloi TOM10	0	7	0	5	7	5	80	24	53.3	3	3	5	5	5	100	21	46.7	90	10	50.0	3.3
C. brassicicola PASS65	0	0	0	0	0	0	0	0	0.0	0	0	0	0	0	0	0	0.0	0	0	0.0	0.0
C. columbiense TOM6	0	0	0	0	0	0	0	0	0.0	0	0	0	0	0	0	0	0.0	0	0	0.0	0.0
C. karsti MAN76	0	0	0	0	0	0	0	0	0.0	0	0	0	0	0	0	0	0.0	0	0	0.0	0.0
C. karsti PASS33	0	0	0	0	0	0	0	0	0.0	0	0	0	0	0	0	0	0.0	0	0	0.0	0.0
C. sydowii PASS35	0	0	0	0	0	0	0	0	0.0	0	0	0	0	0	0	0	0.0	0	0	0.0	0.0
C. gloesporioides s. l. Cg-Sc-A1	0	3	3	3	5	3	100	17	37.8	0	3	3	5	7	80	18	40.0	90	10	38.9	1.1
C. gloesporioides s. l. APL7	0	0	0	0	5	5	40	10	22.2	0	0	0	5	5	40	10	22.2	40	0	22.2	0.0

* Σ(a+b) = sum of infected fruits and their corresponding score scale; n = total number of sampled fruits = 9; z = highest score scale = 9; x = number of infected fruit

PDI (%) = x/n × 100; **PDS*** (%) = Σ(a+b)/n.z × 100; SE= standard error of mean.

Supplementary Table S4 The disease severity scores on strawberry fruits and associated symptoms (Source: Montri et al., 2009)

Score	Symptom description
0	No infection
1	1–2 % of the fruit area shows necrotic lesion or a larger water-soaked lesion surrounding the infection site
3	>2–5 % of the fruit area shows necrotic lesion, acervuli may be present, or water-soaked lesion up to 5 % of the fruit surface
5	>5–15 % of the fruit area shows necrotic lesion, acervuli present, or water-soaked lesion up to 25 % of the fruit surface
7	>15–25 % of the fruit area shows necrotic lesion with acervuli
9	>25 % of the fruit area shows necrosis, lesion often encircling the fruit; abundant acervuli

Supplementary Table S5 Details of GenBank accession numbers of the reference sequences of isolates belonging to the Acutatum species complex (*=type strain)

Species	Culture	ITS	*gapdh*	*chs-1*	*his3*	*act*	*tub2*
C. abscissum	COAD1877*	KP843126	KP843129	KP843132	KP843138	KP843141	KP843135
C. acerbum	CBS128530,ICMP12921,PRJ1199.3*	JQ948459	JQ948790	JQ949120	JQ949450	JQ949780	JQ950110
C. acutatum	CBS112996,ATCC56816,STE-U5292*	JQ005776	JQ948677	JQ005797	JQ005818	JQ005839	JQ005860
C. acutatum	CBS979.69	JQ948400	JQ948731	JQ949061	JQ949391	JQ949721	JQ950051
C. arboricola	CBS144795*,SAG53350-12	MH817944	MH817950	—	—	MH817956	MH817962
C. australe	CBS116478,HKUCC2616*	JQ948455	JQ948786	JQ949116	JQ949446	JQ949776	JQ950106
C. australe	CBS131325,CPC19820	JQ948456	JQ948787	JQ949117	JQ949447	JQ949777	JQ950107
C. brisbanense	CBS292.67,DPI11711*	JQ948291	JQ948621	JQ948952	JQ949282	JQ949612	JQ949942
C. cairnsense	BRIP63642*	KU923672	KU923704	KU923710	KU923722	KU923716	KU923688
C. carthami	SAPA100011*	AB696998	—	—	—	—	AB696992
C. chrysanthemi	IMI364540,CPC	JQ948273	JQ948603	JQ948934	JQ949264	JQ949594	JQ949924
C. cosmi	CBS853.73,PD73/856*	JQ948274	JQ948604	JQ948935	JQ949265	JQ949595	JQ949925
C. costaricense	CBS330.75*	JQ948180	JQ948510	JQ948841	JQ949171	JQ949501	JQ949831
C. costaricense	CBS211.78,IMI309622	JQ948181	JQ948511	JQ948842	JQ949172	JQ949502	JQ949832

C. cuscutae	IMI304802,CPC18873*	JQ948195	JQ948525	JQ948856	JQ949186	JQ949516	JQ949846
C. eriobotryae	GLMC1935*	MF772487	MF795423	MN191653	MN191658	MN191648	MF795428
C. fioriniae	IMI363003,CPC18928	JQ948339	JQ948669	JQ949000	JQ949330	JQ949660	JQ949990
C. fioriniae	CBS128517,ARSEF10222,ERL1257,EHS58*	JQ948292	JQ948622	JQ948953	JQ949283	JQ949613	JQ949943
C. fioriniae	CBS129948,RB128	JQ948344	JQ948674	JQ949005	JQ949335	JQ949665	JQ949995
C. fioriniae	CBS119293,MEP1322	JQ948314	JQ948644	JQ948975	JQ949305	JQ949635	JQ949965
C. godetiae	CBS133.44*	JQ948402	JQ948733	JQ949063	JQ949393	JQ949723	JQ950053
C. godetiae	CBS126522,PD88/472,BBA70345	JQ948411	JQ948742	JQ949072	JQ949402	JQ949732	JQ950062
C. guajavae	IMI350839,CPC18893*	JQ948270	JQ948600	JQ948931	JQ949261	JQ949591	JQ949921
C. indonesiense	CBS127551,CPC14986*	JQ948288	JQ948618	JQ948949	JQ949279	JQ949609	JQ949939
C. javanense	CBS144963*	MH846576	MH846572	MH846573	MH846571	MH846575	MH846574
C. johnstonii	CBS128532,ICMP12926,PRJ1139.3*	JQ948444	JQ948774	JQ949105	JQ949435	JQ949765	JQ950095
C. johnstonii	IMI357027,CPC18924,PRJ1125.005	JQ948443	JQ948773	JQ949104	JQ949434	JQ949764	JQ950094
C. kinghornii	CBS198.35*	JQ948454	JQ948784	JQ949115	JQ949445	JQ949775	JQ950105
C. kniphofiae	CBS143496*	MH107884	MH107998	MH107990	—	MH107975	MH108037
C. laticiphilum	CBS112989,IMI383015,STE-U5303*	JQ948289	JQ948619	JQ948950	JQ949280	JQ949610	JQ949940
C. laticiphilum	CBS129827,CH2	JQ948290	JQ948620	JQ948951	JQ949281	JQ949611	JQ949941
C. lauri	MFLUCC:17-0205*,IT2505_1a	KY514347	KY514344	KY514341	—	KY514338	KY514350
C. limetticola	CBS114.14*	JQ948193	JQ948523	JQ948854	JQ949184	JQ949514	JQ949844
C. lupini	CBS109225,BBA70884*	JQ948155	JQ948485	JQ948816	JQ949146	JQ949476	JQ949806
C. lupini	CBS466.76	JQ948160	JQ948490	JQ948821	JQ949151	JQ949481	JQ949811
C. melonis	CBS159.84*	JQ948194	JQ948524	JQ948855	JQ949185	JQ949515	JQ949845
C. miaoliense	NTUCC20-001-1*	MK908419	MK908470	MK908522	—	MK908573	MK908624
C. nymphaeae as'citri'	ZJUC42, CBS134234	KC293582	KC293742	KY856139	KY856310	KY855974	KC293662
C. nymphaeae	CBS515.78*	JQ948197	JQ948527	JQ948858	JQ949188	JQ949518	JQ949848
C. nymphaeae	CBS516.78, IAM14670	JQ948198	JQ948528	JQ948859	JQ949189	JQ949519	JQ949849
C. nymphaeae,as'citri'	ZJUC41,CBS134233,CGMCC3.15228*	KC293581	KC293741	KY856138	KY856309	KY855973	KC293661
C. paranaense	CBS134729*	KC204992	KC205026	KC205043	KC205004	KC205077	KC205060
C. paxtonii	IMI165753, CPC18868*	JQ948285	JQ948615	JQ948946	JQ949276	JQ949606	JQ949936
C. paxtonii	CBS502.97,LARS58	JQ948286	JQ948616	JQ948947	JQ949277	JQ949607	JQ949937
C. phormii	CBS118194,AR3546*	JQ948446	JQ948777	JQ949107	JQ949437	JQ949767	JQ950097

C. phormii	CBS199.35,DSM1168	JQ948447	JQ948778	JQ949108	JQ949438	JQ949768	JQ950098
C. pyricola	CBS128531, ICMP12924,PRJ977.1*	JQ948445	JQ948776	JQ949106	JQ949436	JQ949766	JQ950096
C. rhombiforme	CBS129953, PT250, RB011*	JQ948457	JQ948788	JQ949118	JQ949448	JQ949778	JQ950108
C. rhombiforme	CBS131322, DAOM233253, C10, MS1L34	JQ948458	JQ948789	JQ949119	JQ949449	JQ949779	JQ950109
C. roseum	CBS145754*	MK903611	MK903603	—	—	MK903604	MK903607
C. salicis	CBS607.94*	JQ948460	JQ948791	JQ949121	JQ949451	JQ949781	JQ950111
C. salicis	CBS191.56	JQ948461	JQ948792	JQ949122	JQ949452	JQ949782	JQ950112
C. schimae sp. nov.	LC13880, NN046984*	MZ595885	MZ664105	MZ799347	MZ673905	MZ664183	MZ674003
C. schimae sp. nov.	LC13881, NN047247	MZ595887	MZ664106	MZ799348	MZ673907	MZ664185	MZ674005
C. scovillei	CBS126529, PD94/921-3, BBA70349*	JQ948267	JQ948597	JQ948928	JQ949258	JQ949588	JQ949918
C. scovillei	CBS126530,PD94/921-4	JQ948268	JQ948598	JQ948929	JQ949259	JQ949589	JQ949919
C. scovillei	CBS120708,HKUCC10893,Mj6	JQ948269	JQ948599	JQ948930	JQ949260	JQ949590	JQ949920
C. simmondsii	CBS122122,BRIP28519*	JQ948276	JQ948606	JQ948937	JQ949267	JQ949597	JQ949927
C. simmondsii	CBS295.67,DPI16518	JQ948278	JQ948608	JQ948939	JQ949269	JQ949599	JQ949929
C. sloanei	IMI364297,CPC18929*	JQ948287	JQ948617	JQ948948	JQ949278	JQ949608	JQ949938
C. subsalicis sp. nov.	LC13863,CQ1168*	MZ852849	—	MZ799346	MZ673836	MZ664128	MZ673953
C. tamarilloi	CBS129814,T.A.6*	JQ948184	JQ948514	JQ948845	JQ949175	JQ949505	JQ949835
C. tamarilloi	CBS129811,T.A.3	JQ948185	JQ948515	JQ948846	JQ949176	JQ949506	JQ949836
C. walleri	CBS125472,BMT(HL)19*	JQ948275	JQ948605	JQ948936	JQ949266	JQ949596	JQ949926
C. wanningense	CGMCC3.18936*	MG830462	MG830318	MG830302	—	MG830270	MG830286
Monilochaetes infuscans	CBS869.96	JQ005780	JX546612	JQ005801	JQ005822	JQ005843	JQ005864

Supplementary Table S6 Details of GenBank accession numbers of the additional reference sequences of isolates belonging to the *C. fioriniae*, *C. godetiae* and *C. nymphaeae* species (*=type strain)

**Species**	**Culture**	**ITS**	***gapdh***	***chs-1***	***his3***	***act***	***tub2***
C. fioriniae	IMI 324996	JQ948301	JQ948631	JQ948962	JQ949292	JQ949622	JQ949952
C. fioriniae	CBS 126526	JQ948323	JQ948653	JQ948984	JQ949314	JQ949644	JQ949974
C. fioriniae	CBS 124958	JQ948306	JQ948636	JQ948967	JQ949297	JQ949627	JQ949957

C. fioriniae	CBS 119292	JQ948313	JQ948643	JQ948974	JQ949304	JQ949634	JQ949964
C. fioriniae	CBS 125396	JQ948299	JQ948629	JQ948960	JQ949290	JQ949620	JQ949950
C. fioriniae	IMI363003	JQ948339	JQ948669	JQ949000	JQ949330	JQ949660	JQ949990
C. fioriniae	CBS128517*	JQ948292	JQ948622	JQ948953	JQ949283	JQ949613	JQ949943
C. fioriniae	CBS129948	JQ948344	JQ948674	JQ949005	JQ949335	JQ949665	JQ949995
C. fioriniae	CBS119293	JQ948314	JQ948644	JQ948975	JQ949305	JQ949635	JQ949965
C. fioriniae	CBS 235.49	JQ948325	JQ948655	JQ948986	JQ949316	JQ949646	JQ949976
C. fioriniae	CBS 127601	JQ948311	JQ948641	JQ948972	JQ949302	JQ949632	JQ949962
C. fioriniae	CBS 167.86	JQ948324	JQ948654	JQ948985	JQ949315	JQ949645	JQ949975
C. fioriniae	CBS 129946	JQ948342	JQ948672	JQ949003	JQ949333	JQ949663	JQ949993
C. fioriniae	CBS 126509	JQ948316	JQ948646	JQ948977	JQ949307	JQ949637	JQ949967
C. fioriniae	CBS 125956	JQ948321	JQ948651	JQ948982	JQ949312	JQ949642	JQ949972
C. fioriniae	CBS 293.67	JQ948310	JQ948640	JQ948971	JQ949301	JQ949631	JQ949961
C. fioriniae	CBS 200.35	JQ948293	JQ948623	JQ948954	JQ949284	JQ949614	JQ949944
C. fioriniae	CBS 490.92	JQ948326	JQ948656	JQ948987	JQ949317	JQ949647	JQ949977
C. fioriniae	CBS 124962	JQ948319	JQ948649	JQ948980	JQ949310	JQ949640	JQ949970
C. fioriniae	CBS 124963	JQ948320	JQ948650	JQ948981	JQ949311	JQ949641	JQ949971
C. fioriniae	CBS 126508	JQ948315	JQ948645	JQ948976	JQ949306	JQ949636	JQ949966
C. fioriniae	CBS 119186	JQ948312	JQ948642	JQ948973	JQ949303	JQ949633	JQ949963
C. fioriniae	CBS 127537	JQ948318	JQ948648	JQ948979	JQ949309	JQ949639	JQ949969
C. fioriniae	CBS 129916	JQ948317	JQ948647	JQ948978	JQ949308	JQ949638	JQ949968
C. fioriniae	CBS 129947	JQ948343	JQ948673	JQ949004	JQ949334	JQ949664	JQ949994
C. godetiae	CBS 796.72	JQ948407	JQ948738	JQ949068	JQ949398	JQ949728	JQ950058
C. godetiae	IMI 351248	JQ948433	JQ948764	JQ949094	JQ949424	JQ949754	JQ950084
C. godetiae	CBS 193.32	JQ948415	JQ948746	JQ949076	JQ949406	JQ949736	JQ950066
C. godetiae	CBS133.44*	JQ948402	JQ948733	JQ949063	JQ949393	JQ949723	JQ950053
C. godetiae	CBS131332	JQ948429	JQ948760	JQ949090	JQ949420	JQ949750	JQ950080
C. godetiae	CBS125972	JQ948416	JQ948747	JQ949077	JQ949407	JQ949737	JQ950067
C. godetiae	CBS126376	JQ948417	JQ948748	JQ949078	JQ949408	JQ949738	JQ950068
C. godetiae	CBS126516	JQ948418	JQ948749	JQ949079	JQ949409	JQ949739	JQ950069
C. godetiae	IMI345026	JQ948424	JQ948755	JQ949085	JQ949415	JQ949745	JQ950075

C. godetiae	CBS125974	JQ948419	JQ948750	JQ949080	JQ949410	JQ949740	JQ950070
C. godetiae	CBS126503	JQ948420	JQ948751	JQ949081	JQ949411	JQ949741	JQ950071
C. godetiae	IMI351253	JQ948421	JQ948752	JQ949082	JQ949412	JQ949742	JQ950072
C. godetiae	CBS171.59	JQ948405	JQ948736	JQ949066	JQ949396	JQ949726	JQ950056
C. godetiae	CBS131331	JQ948404	JQ948735	JQ949065	JQ949395	JQ949725	JQ950055
C. godetiae	IMI362149b	JQ948427	JQ948758	JQ949088	JQ949418	JQ949748	JQ950078
C. godetiae	CBS126522	JQ948411	JQ948742	JQ949072	JQ949402	JQ949732	JQ950062
C. godetiae	CBS129934	JQ948431	JQ948762	JQ949092	JQ949422	JQ949752	JQ950082
C. godetiae	IMI376331	JQ948409	JQ948740	JQ949070	JQ949400	JQ949730	JQ950060
C. godetiae	IMI381927	JQ948438	JQ948769	JQ949099	JQ949429	JQ949759	JQ950089
C. godetiae	CBS862.7	JQ948437	JQ948768	JQ949098	JQ949428	JQ949758	JQ950088
C. godetiae	CBS129951	JQ948430	JQ948761	JQ949091	JQ949421	JQ949751	JQ950081
C. godetiae	CBS129917	JQ948441	JQ948772	JQ949102	JQ949432	JQ949762	JQ950092
C. godetiae	CBS129809	JQ948439	JQ948770	JQ949100	JQ949430	JQ949760	JQ950090
C. godetiae	CBS129816	JQ948440	JQ948771	JQ949101	JQ949431	JQ949761	JQ950091
C. godetiae	CBS127561	JQ948442	JQ948773	JQ949103	JQ949433	JQ949763	JQ950093
C. nymphaeae	IMI 360386	JQ948206	JQ948536	JQ948867	JQ949197	JQ949527	JQ949857
C. nymphaeae	CBS515.78*	JQ948197	JQ948527	JQ948858	JQ949188	JQ949518	JQ949848
C. nymphaeae	CBS516.78	JQ948198	JQ948528	JQ948859	JQ949189	JQ949519	JQ949849
C. nymphaeae	CBS127612	JQ948230	JQ948560	JQ948891	JQ949221	JQ949551	JQ949881
C. nymphaeae	CBS112202	JQ948234	JQ948564	JQ948895	JQ949225	JQ949555	JQ949885
C. nymphaeae	IMI299103	JQ948231	JQ948561	JQ948892	JQ949222	JQ949552	JQ949882
C. nymphaeae	CBS126383	JQ948221	JQ948551	JQ948882	JQ949212	JQ949542	JQ949872
C. nymphaeae	CBS100064	JQ948224	JQ948554	JQ948885	JQ949215	JQ949545	JQ949875
C. nymphaeae	CBS129935	JQ948227	JQ948557	JQ948888	JQ949218	JQ949548	JQ949878
C. nymphaeae	CBS361.79	JQ948248	JQ948578	JQ948909	JQ949239	JQ949569	JQ949899
C. nymphaeae	CBS126382	JQ948220	JQ948550	JQ948881	JQ949211	JQ949541	JQ949871
C. nymphaeae	CBS126511	JQ948222	JQ948552	JQ948883	JQ949213	JQ949543	JQ949873
C. nymphaeae	IMI345053	JQ948239	JQ948569	JQ948900	JQ949230	JQ949560	JQ949890
C. nymphaeae	CBS125958	JQ948245	JQ948575	JQ948906	JQ949236	JQ949566	JQ949896
C. nymphaeae	CBS125959	JQ948246	JQ948576	JQ948907	JQ949237	JQ949567	JQ949897

C. nymphaeae	CBS126504	JQ948265	JQ948595	JQ948926	JQ949256	JQ949586	JQ949916
C. nymphaeae	IMI364856	JQ948244	JQ948574	JQ948905	JQ949235	JQ949565	JQ949895
C. nymphaeae	CBS129945	JQ948201	JQ948531	JQ948862	JQ949192	JQ949522	JQ949852
C. nymphaeae	CBS231.49	JQ948202	JQ948532	JQ948863	JQ949193	JQ949523	JQ949853
C. nymphaeae	CBS482.82	JQ948213	JQ948543	JQ948874	JQ949204	JQ949534	JQ949864
C. nymphaeae	CBS115408	JQ948212	JQ948542	JQ948873	JQ949203	JQ949533	JQ949863
C. nymphaeae	CBS112992	JQ948207	JQ948537	JQ948868	JQ949198	JQ949528	JQ949858
C. nymphaeae	CBS113002	JQ948208	JQ948538	JQ948869	JQ949199	JQ949529	JQ949859
C. nymphaeae	CBS114188	JQ948214	JQ948544	JQ948875	JQ949205	JQ949535	JQ949865
C. nymphaeae	CBS158.27	JQ948215	JQ948545	JQ948876	JQ949206	JQ949536	JQ949866
C. nymphaeae,as'*citri*'	CBS134233*	KC293581	KC293741	KY856138	KY856309	KY855973	KC293661
C. tamarilloi	CBS129814*	JQ948184	JQ948514	JQ948845	JQ949175	JQ949505	JQ949835
C. tamarilloi	CBS129811	JQ948185	JQ948515	JQ948846	JQ949176	JQ949506	JQ949836
C. acutatum	CBS112996*	JQ005776	JQ948677	JQ005797	JQ005818	JQ005839	JQ005860
C. acutatum	CBS979.69	JQ948400	JQ948731	JQ949061	JQ949391	JQ949721	JQ950051
Monilochaetes infuscans	CBS869.96	JQ005780	JX546612	JQ005801	JQ005822	JQ005843	JQ005864

Supplementary Table S7 Details of GenBank accession numbers of the reference sequences of isolates belonging to the Boninense species complex (*=type strain)

**Species**	**Culture**	**ITS**	***gapdh***	***chs-1***	***his3***	***act***	***tub2***
C. annellatum	CBS129826,CH1*	JQ005222	JQ005309	JQ005396	JQ005483	JQ005570	JQ005656
C. beeveri	CBS128527,ICMP18594*	JQ005171	JQ005258	JQ005345	JQ005432	JQ005519	JQ005605
C. beeveri	NN004142	MZ595881	MZ664082	MZ799277	MZ673901	MZ664179	—
C. boninense	CBS123755,MAFF305972*	JQ005153	JQ005240	JQ005327	JQ005414	JQ005501	JQ005588
C. brasiliense	CBS128501,ICMP18607,PAS12*	JQ005235	JQ005322	JQ005409	JQ005496	JQ005583	JQ005669
C. brasiliense	CBS128528,ICMP18606,PAS10	JQ005234	JQ005321	JQ005408	JQ005495	JQ005582	JQ005668
C. brassicicola	CBS101059,LYN16331*	JQ005172	JQ005259	JQ005346	JQ005433	JQ005520	JQ005606
C. bromeliacearum sp. nov.	LC0951*	MZ595832	MZ664077	MZ799267	MZ673843	MZ664130	MZ673956

C. bromeliacearum sp. nov.	LC13854,LC0951-1	MZ595833	MZ664078	MZ799268	MZ673844	MZ664131	OK360930
C. bromeliacearum sp. nov.	LC13855,LC0951-2	MZ595834	MZ664079	MZ799269	MZ673845	MZ664132	OK360931
C. bromeliacearum sp. nov.	LC13856,LC0951-3	MZ595835	MZ664080	MZ799270	MZ673846	MZ664133	OK360932
C. camelliae-japonicae	CGMCC3.18118*,LC6416	KX853165	KX893584	MZ799271	MZ673859	KX893576	KX893580
C. catinaense	CBS142417*,CPC27978	KY856400	KY856224	KY856136	KY856307	KY855971	KY856482
C. chamaedoreae sp. nov.	LC13867,NN052884	MZ595889	MZ664083	MZ799273	MZ673909	MZ664187	MZ674007
C. chamaedoreae sp. nov.	LC13868,NN052885*	MZ595890	MZ664084	MZ799274	MZ673910	MZ664188	MZ674008
C. chamaedoreae sp. nov.	LC13869,NN052890	MZ595891	MZ664086	MZ799275	MZ673911	MZ664189	MZ674009
C. chamaedoreae sp. nov.	LC13870,NN052891	MZ595892	MZ664085	MZ799276	MZ673912	MZ664190	MZ674010
C. citricola	ZJUC34,CBS134228,CGMCC3.15227*	KC293576	KC293736	—	—	KC293616	KC293656
C. chongqingense	CS0612*	MG602060	MG602022	MT976117	—	MT976107	MG602044
C. colombiense	CBS129818*	JQ005174	JQ005261	JQ005348	JQ005435	JQ005522	JQ005608
C. condaoense	CBS134299*	MH229914	MH229920	MH229926	MH229927	—	MH229923
C. constrictum	CBS128504,ICMP12941*	JQ005238	JQ005325	JQ005412	JQ005499	JQ005586	JQ005672
C. constrictum	CBS128503,ICMP12936	JQ005237	JQ005324	JQ005411	JQ005498	JQ005585	JQ005671
C. cymbidiicola	IMI347923*	JQ005166	JQ005253	JQ005340	JQ005427	JQ005514	JQ005600
C. cymbidiicola	CBS128543,ICMP18584	JQ005167	JQ005254	JQ005341	JQ005428	JQ005515	JQ005601
C. cymbidiicola	CBS123757,MAFF306100	JQ005168	JQ005255	JQ005342	JQ005429	JQ005516	JQ005602
C. dacrycarpi	CBS130241,ICMP19107*	JQ005236	JQ005323	JQ005410	JQ005497	JQ005584	JQ005670
C. diversum sp. nov.	LC11292,CQ775*	MZ595844	MZ664081	MZ799272	MZ673864	MZ664142	MZ673965
C. doitungense	MFLUCC14-0128*	MF448524	MH049480	—	—	MH376385	MH351277
C. feijoicola	CBS144633*	MK876413	MK876475	—	—	MK876466	MK876507
C. feijoicola	CPC34245	MK876414	MK876474	MK876471	MK876477	MK876465	MK876506
C. hippeastri	CBS125376,CSSG1*	JQ005231	JQ005318	JQ005405	JQ005492	JQ005579	JQ005665
C. hippeastri	CBS241.78,IMI304052	JQ005232	JQ005319	JQ005406	JQ005493	JQ005580	JQ005666
C. karsti	CBS861.72	JQ005184	JQ005271	JQ005358	JQ005445	JQ005532	JQ005618
C. karsti	CBS106.91	JQ005220	JQ005307	JQ005394	JQ005481	JQ005568	JQ005654
C. karsti	CBS110779	JQ005198	JQ005285	JQ005372	JQ005459	JQ005546	JQ005632
C. limonicola	CBS142409,CPC27861	KY856471	KY856295	KY856212	KY856387	KY856044	KY856553
C. limonicola	CBS142410*,CPC31141	KY856472	KY856296	KY856213	KY856388	KY856045	KY856554
C. novae-zelandiae	CBS128505,ICMP12944*	JQ005228	JQ005315	JQ005402	JQ005489	JQ005576	JQ005662

C. novae-zelandiae	CBS130240,ICMP12064	JQ005229	JQ005316	JQ005403	JQ005490	JQ005577	JQ005663
C. oncidii	CBS129828*	JQ005169	JQ005256	JQ005343	JQ005430	JQ005517	JQ005603
C. oncidii	CBS130242	JQ005170	JQ005257	JQ005344	JQ005431	JQ005518	JQ005604
C. parsonsiae	CBS128525,ICMP18590*	JQ005233	JQ005320	JQ005407	JQ005494	JQ005581	JQ005667
C. petchii	CBS378.94*	JQ005223	JQ005310	JQ005397	JQ005484	JQ005571	JQ005657
C. petchii	CBS118193,AR3658	JQ005227	JQ005314	JQ005401	JQ005488	JQ005575	JQ005661
C. petchii	CBS125957,NB145	JQ005226	JQ005313	JQ005400	JQ005487	JQ005574	JQ005660
C. phyllanthi	CBS175.67,MACS271*	JQ005221	JQ005308	JQ005395	JQ005482	JQ005569	JQ005655
C. torulosum	CBS128544,ICMP18586*	JQ005164	JQ005251	JQ005338	JQ005425	JQ005512	JQ005598
C. torulosum	CBS102667	JQ005165	JQ005252	JQ005339	JQ005426	JQ005513	JQ005599
C. watphraense	MFLUCC14-0123*	MF448523	MH049479	—	—	MH376384	MH351276
Colletotrichum sp. *CBS123921*	MAFF238642	JQ005163	JQ005250	JQ005337	JQ005424	JQ005511	JQ005597
Monilochaetes infuscans	CBS869.96	JQ005780	JX546612	JQ005801	JQ005822	JQ005843	JQ005864

Supplementary Table S8 Details of GenBank accession numbers of the reference sequences of isolates belonging to the Gloeosporioides species complex (*=type strain)

Species	Culture	ITS	*gapdh*	*chs-1*	*his3*	*act*	*tub2*
C. aenigma	ICMP18608*	JX010244	JX010044	JX009774	—	JX009443	JX010389
C. aeschynomenes	ICMP17673*,ATCC201874	JX010176	JX009930	JX009799	—	JX009483	JX010392
C. alatae	CBS304.67*,ICMP17919	JX010190	JX009990	JX009837	—	JX009471	JX010383
C. alienum	ICMP12071*	JX010251	JX010028	JX009882	—	JX009572	JX010411
C. aotearoa	ICMP18537*	JX010205	JX010005	JX009853	—	JX009564	JX010420
C. arecicola	CGMCC3.19667*	MK914635	MK935455	MK935541	—	MK935374	MK935498
C. artocarpicola	MFLUCC18-1167*	MN415991	MN435568	MN435569	—	MN435570	MN435567
C. asianum	ICMP18580*,CBS130418	FJ972612	JX010053	JX009867	—	JX009584	JX010406
C. australianum	VPRI43075*	MG572138	MG572127	MW091987	—	MN442109	MG572149
C. camelliae	CGMCC3.14925,LC1364*	KJ955081	KJ954782	MZ799255	MZ673847	KJ954363	KJ955230
C. changpingense	CGMCC3.17582*,SA0016,MFLUCC15-0022	KP683152	MZ664048	KP852449	—	KP683093	MZ673952

C. chiangmaiense	MFLUCC18-0945*	MW346499	MW548592	MW623653	—	MW655578	—
C. chrysophilum	URM7368,CMM4268*	KX094252	KX094183	KX094083	—	KX093982	KX094285
C. cigarro	ICMP18539*	JX010230	JX009966	JX009800	MZ673837	JX009523	JX010434
C. clidemiae	ICMP18658*	JX010265	JX009989	JX009877	—	JX009537	JX010438
C. cobbittiense	BRIP66219*	MH087016	MH094133	MH094135	MH094136	MH094134	MH094137
C. conoides	CGMCC3.17615,CAUG17,LC6226*	KP890168	KP890162	KP890156	—	KP890144	KP890174
C. cordylinicola	MFLUCC090551*,ICMP18579	JX010226	JX009975	JX009864	—	HM470235	JX010440
C. dracaenigenum	MFLUCC19-0430*	MN921250	MT215577	MT215575	—	MT313686	—
C. endophyticum	MFLUCC13-0418,LC0324*	KC633854	KC832854	MZ799261	MZ673839	KF306258	MZ673954
C. fructicola	ICMP18581*,CBS130416	JX010165	JX010033	JX009866	—	FJ907426	JX010405
C. fructivorum	Coll1414,BPI884103,CBS133125*	JX145145	MZ664047	MZ799259	—	MZ664126	JX145196
C. gloeosporioides	IMI356878*,ICMP17821,CBS112999	JX010152	JX010056	JX009818	JQ005413	JX009531	JX010445
C. grevilleae	CBS132879,CPC15481*	KC297078	KC297010	KC296987	KC297056	KC296941	KC297102
C. grossum	CGMCC3.17614,CAUG7,LC6227*	KP890165	KP890159	KP890153	—	KP890141	KP890171
C. hebeiense	MFLUCC13–0726*	KF156863	KF377495	KF289008	—	KF377532	KF288975
C. hederiicola	MFLU15-0689*	MN631384	—	MN635794	—	MN635795	—
C. helleniense	CBS142418,CPC26844*	KY856446	KY856270	KY856186	KY856361	KY856019	KY856528
C. henanense	LC3030,CGMCC3.17354,LF238*	KJ955109	KJ954810	MZ799256	MZ673835	KM023257	KJ955257
C. horii	NBRC7478*,ICMP10492,MTCC10841	GQ329690	GQ329681	JX009752	—	JX009438	JX010450
C. hystricis	CBS142411,CPC28153*	KY856450	KY856274	KY856190	KY856365	KY856023	KY856532
C. jiangxiense	CGMCC3.17361*,LC3266,LF488	KJ955149	KJ954850	MZ799257	—	KJ954427	OK236389
C. kahawae	IMI319418*,ICMP17816	JX010231	JX010012	JX009813	MZ673838	JX009452	JX010444
C. makassarense	CBS143664*	MH728812	MH728820	MH805850	—	MH781480	MH846563
C. musae	CBS116870*,ICMP19119,MTCC11349	JX010146	JX010050	JX009896	—	JX009433	HQ596280
C. nupharicola	CBS470.96*,ICMP18187	JX010187	JX009972	JX009835	—	JX009437	JX010398
C. pandanicola	MFLUCC17-0571*	MG646967	MG646934	MG646931	—	MG646938	MG646926
C. perseae	CBS141365*,GA100	KX620308	KX620242	MZ799260	—	KX620145	KX620341
C. proteae	CBS132882*,CPC14859	KC297079	KC297009	KC296986	KC297045	KC296940	KC297101
C. pseudotheobromicola	MFLUCC18–1602*	MH817395	MH853675	MH853678	—	MH853681	MH853684
C. psidii	CBS145.29*,ICMP19120	JX010219	JX009967	JX009901	—	JX009515	JX010443
C. queenslandicum	ICMP1778*	JX010276	JX009934	JX009899	—	JX009447	JX010414

C. rhexiae	Coll1026,BPI884112,CBS133134*	JX145128	MZ664046	MZ799258	MZ673834	MZ664127	JX145179
C. salsolae	ICMP19051*	JX010242	JX009916	JX009863	—	JX009562	JX010403
C. siamense	ICMP18578*,CBS130417	JX010171	JX009924	JX009865	—	FJ907423	JX010404
C. syzygiicola	DNCL021,MFLUCC10-0624*	KF242094	KF242156	—	—	KF157801	KF254880
C. tainanense	CBS143666*	MH728818	MH728823	MH805845	—	MH781475	MH846558
C. temperatum	CBS133122*,Coll883,BPI884100	JX145159	MZ664045	MZ799254	MZ673833	MZ664125	JX145211
C. theobromicola	CBS124945*,ICMP18649	JX010294	JX010006	JX009869	—	JX009444	JX010447
C. ti	ICMP4832*	JX010269	JX009952	JX009898	—	JX009520	JX010442
C. tropicale	CBS124949*,ICMP18653,MTCC11371	JX010264	JX010007	JX009870	MZ673832	JX009489	JX010407
C. viniferum	GZAAS5.08601*,yg1	JN412804	JN412798	—	—	JN412795	—
C. wuxiense	CGMCC3.17894*	KU251591	KU252045	KU251939	—	KU251672	KU252200
C. xanthorrhoeae	BRIP45094*,ICMP17903,CBS127831	JX010261	JX009927	JX009823	—	JX009478	JX010448
C. xishuangbannaense	MFLUCC19-0107*	MW346469	MW537586	MW660832	—	MW652294	—
C. yulongense	CFCC50818*	MH751507	MK108986	MH793605	—	MH777394	MK108987
Monilochaetes infuscans	CBS869.96	JQ005780	JX546612	JQ005801	JQ005822	JQ005843	JQ005864
